# Design, synthesis, and biological evaluation of derivatives from p-toluic hydrazide: lead compound C17 exhibits membrane-disrupting and anti-biofilm activities against *Staphylococcus aureus* and other gram-positive bacteria

**DOI:** 10.3389/fchem.2026.1825790

**Published:** 2026-05-14

**Authors:** Yaguang Liu, Lianzhi Hu, Binbin Liu, Zheng Qu

**Affiliations:** The Second Hospital of Qinhuangdao, Pharmacy Department, Qinhuangdao, China

**Keywords:** anti biofilm, anti inflammatory, antibacterial activity, drug-likeness, Schiff base

## Abstract

**Introduction:**

Bacterial drug resistance and biofilm-associated infections pose major therapeutic challenges. Schiff base derivatives have attracted attention as potential antimicrobial agents. This study aimed to design and synthesize a series of Schiff base derivatives from p-toluic hydrazide and evaluate their antibacterial, anti-biofilm, and anti-inflammatory properties.

**Methods:**

Eighteen Schiff base derivatives were synthesized from p-toluic hydrazide. Antibacterial activity was screened against a panel of Gram-positive bacteria, including *Staphylococcus aureus* ATCC 29213, methicillin-resistant *S. aureus* (MRSA) ATCC 43300, Bacillus subtilis, Bacillus cereus, Listeria monocytogenes, and Enterococcus faecalis, by determining minimum inhibitory concentrations (MICs). The most active compound, **C17**, was further evaluated for hemolytic activity (against red blood cells), cytotoxicity (VERO cells), and mechanistic effects (membrane depolarization, membrane permeability, reactive oxygen species accumulation, protein leakage). Biofilm inhibition and eradication assays, anti-inflammatory activity (LPS-induced IL-6 and TNF-α production in RAW 264.7 macrophages), and pharmacokinetic properties (plasma protein binding, lipophilicity logD7.4, metabolic stability in liver microsomes) were also assessed.

**Results:**

Compound **C17** exhibited potent activity against multiple Gram-positive bacteria with MIC values of 16–32 μg/mL. It showed no hemolysis up to 256 μg/mL and no significant cytotoxicity in VERO cells, indicating good biocompatibility. Mechanistically, **C17** induced membrane depolarization, increased membrane permeability, promoted ROS accumulation, and caused protein leakage, confirming membrane disruption as its primary bactericidal action. **C17** significantly inhibited *S. aureus* biofilm formation (92% inhibition at 8× MIC) and eradicated preformed biofilms (69% eradication at 8× MIC). Furthermore, **C17** suppressed LPS-induced production of IL-6 and TNF-α in RAW 264.7 macrophages, demonstrating anti-inflammatory activity. Pharmacokinetic profiling revealed high plasma protein binding (89.3%), moderate lipophilicity (logD7.4 = 3.14), and acceptable metabolic stability (T_1_/_2_ = 52.98 min in liver microsomes).

**Discussion:**

Collectively, **C17** is a promising lead candidate for treating biofilm-associated and drug-resistant Gram-positive bacterial infections. Its multi-faceted action—direct membrane disruption, biofilm inhibition/eradication, and anti-inflammatory effects–combined with favorable biocompatibility and acceptable pharmacokinetics, warrants further development and in-depth investigation.

## 1 Introduction

Bacterial infectious diseases have long posed a significant threat to human health. Since the discovery of penicillin, the widespread application of antibiotics has greatly improved the treatment outcomes of infectious diseases and saved countless lives (Baran et al., 2023; Deusenbery et al., 2021). However, with the overuse and even misuse of antibiotics in medical, agricultural, and livestock settings, bacterial antibiotic resistance has evolved into a global public health crisis (Diallo et al., 2020; Lyu et al., 2023). In this evolutionary race between humans and microbes, the performance of Gram-positive bacteria, particularly *Staphylococcus aureus* and its drug-resistant form methicillin-resistant *S*. *aureus* MRSA, is especially concerning (Barbosa et al., 2021; Kaushik et al., 2024). These pathogens can cause a wide range of illnesses, from minor skin infections to life-threatening conditions such as bacteremia, pneumonia, and endocarditis. More importantly, through mechanisms such as genetic mutation and horizontal gene transfer, they rapidly develop resistance to multiple antibiotics (Guo et al., 2020; Lakhundi and Zhang, 2018). MRSA exhibits widespread resistance to first-line antibiotics including β-lactams, macrolides, and fluoroquinolones, resulting in severely limited treatment options and increasing reliance on last-resort drugs such as vancomycin and linezolid (Mühlberg et al., 2020; Pereyre et al., 2016). This severe resistance situation not only substantially increases medical costs and treatment difficulty but also directly leads to higher patient mortality. Therefore, there is an urgent need to develop novel antibacterial agents with new chemical structures, unique mechanisms of action, and the ability to effectively circumvent existing resistance pathways.

The exploration of novel antibacterial agents among Schiff base compounds has become an important research direction. These compounds are formed through a nucleophilic addition-elimination reaction between primary amines and aldehydes or ketones. The imine group (-C=N-) in their structure exhibits unique electronic distribution characteristics, coordination capacity, and chemical flexibility (Es-Sounni et al., 2024; Imran et al., 2014; Liu et al., 2023). Schiff bases are not only relatively simple to synthesize with high yields, but more importantly, the selection of different amine and aldehyde/ketone precursors enables convenient construction of structurally diverse compound libraries, providing an excellent platform for systematic structure-activity relationship studies (Mistri and Mondal, 2024; Udhayakumari and Inbaraj, 2020). Extensive research has demonstrated that Schiff bases and their metal complexes exhibit broad biological activities. Hydrazide-based Schiff bases often display enhanced bioactivity, likely due to inherent pharmacological properties of the acyl group that synergize with the imine functionality (Reshma et al., 2022; Şenol et al., 2023). As an important hydrazide derivative, p-toluic hydrazide contains a highly nucleophilic hydrazine group that reacts efficiently with aldehydes to form Schiff bases, while the toluene moiety provides moderate hydrophobicity and steric hindrance. These characteristics have drawn significant attention to p-toluic hydrazide-derived Schiff bases in medicinal chemistry research (Mbaveng et al., 2016; Zhao et al., 2021). Although p-toluic hydrazide has been investigated as a preservative in the food industry besides its potential pharmaceutical applications, and such compounds may exert antibacterial effects by disrupting bacterial membrane structures, their precise mechanisms of action and structure-activity relationships still require in-depth exploration (Bansal et al., 2021; Parupalli et al., 2022).

**FIGURE 1:**
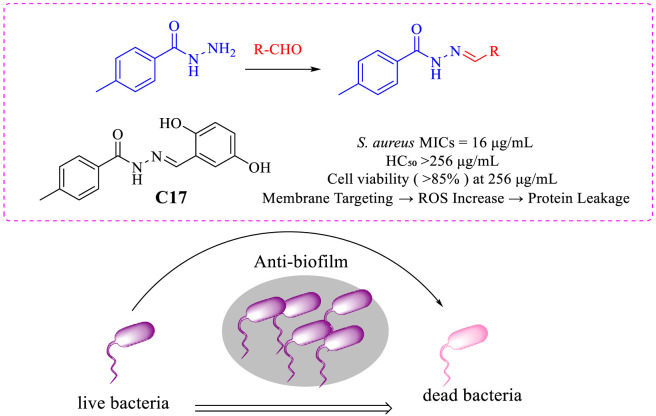
Antibacterial mechanism diagram of Schiff base derivatives.

Based on the aforementioned research background and rationale, this study selected p-toluic hydrazide as a uniform amine component and condensed it with various structurally diverse aldehydes to successfully design and synthesize a series of novel Schiff base derivatives. This strategy allowed us to retain the potential bioactive advantages conferred by the hydrazide moiety while systematically modulating the electronic effects, lipophilicity, and spatial configuration of the final products by introducing diverse aldehyde components—including aromatic aldehydes, aliphatic aldehydes, and substituted aromatic aldehydes—thereby exploring the influence of these factors on antibacterial activity. This work aims not only to report a promising antibacterial lead compound, but also demonstrates a systematic antibacterial drug research pipeline encompassing synthesis, activity screening, mechanism investigation, and preliminary druggability evaluation (Figure 1). It is expected to provide both new candidate compounds and scientific insights for addressing the increasingly serious challenge of bacterial drug resistance.

## 2 Results and discussion

### 2.1 Chemical synthesis

**SCHEME 1:**
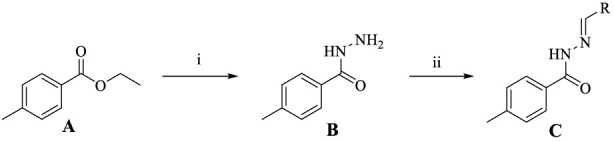
Design and synthesis of Schiff base derivatives. **(A)** Ethyl 4-methylbenzoate; **(B)** 4-Methylbenzohydrazide; **(C)** Schiff base derivatives. Conditions and reagents: (i) Ethanol, NH_2_NH_2_⋅H_2_O, reflux, 88% yield; (ii) Ethanol, R‐CHO, reflux, 80–92% yield.

In this study, ethyl p-methylbenzoate **A** was used as the starting material, and a series of novel *N*′-substituted methylene p-toluic hydrazide derivatives **C1**–**C18** were successfully synthesized according to previously reported methods. The general synthetic route is illustrated in Scheme 1 (Shen et al., 2022; Zhong et al., 2022). The key intermediate p-toluic hydrazide B was synthesized from ethyl p-methylbenzoate **A** via hydrazinolysis in 88% yield. Condensation of **B** with various aldehydes under reflux in ethanol afforded the target Schiff base derivatives **C1**–**C18** in overall yields of 80%–92% after recrystallization. The structures were confirmed by ^1^H NMR, ^13^C NMR, and HRMS. All compounds displayed the characteristic imine proton signal (–CH=N–) in the range δ 7.50–8.50 ppm and the hydrazide NH signal between δ 10.0–11.0 ppm, confirming successful hydrazone formation. HRMS showed [M + H]^+^ ions matching the calculated molecular formulas (see representative data in Experimental section).

### 2.2 Determination of minimum inhibitory concentration

Reportedly, certain Schiff base derivatives demonstrate promising antibacterial potential *in vitro* (Maia et al., 2022; Xia et al., 2015). Therefore, this study evaluated the minimum inhibitory concentrations (MICs) of the synthesized target compounds against a panel of bacterial strains using the broth microdilution method. As shown in Table 1, most derivatives showed no activity up to 256 μg/mL, with the exception of salicylaldehyde-derived Schiff bases (**C13**–**C17**). Among these, compound **C17** exhibited the most potent and consistent activity against all tested Gram-positive strains, with MIC values of 16 μg/mL against *S. aureus* (including MRSA) and 16–32 μg/mL against other Gram-positive pathogens such as *B. cereus*, *L. monocytogenes*, and *E. faecalis* (Table 2). In contrast, **C17** showed only weak activity against Gram-negative bacteria (MIC 256 μg/mL), indicating a Gram-positive selective profile. This selectivity is likely attributable to the presence of the outer membrane in Gram-negative bacteria, which limits access of hydrophobic compounds like **C17**. Notably, among all tested derivatives, the salicylaldehyde-derived compounds (**C13**–**C17**) showed the highest activity, suggesting that the ortho-hydroxyl group on the aromatic ring plays a critical role in antibacterial potency. Among these, **C17** (2,5-dihydroxy) exhibited the lowest MIC (16 μg/mL), whereas **C13** (5-tert-butyl-2-hydroxy), **C14** (5-nitro-2-hydroxy), **C15** (5-methyl-2-hydroxy), and **C16** (5-chloro-2-hydroxy) showed much higher MICs (128–256 μg/mL). This indicates that a second hydroxyl group at the 5-position is favorable for activity, while bulky, electron-withdrawing, or small alkyl substituents reduce potency. The additional para-hydroxyl group in **C17** likely enhances hydrogen bonding with membrane phospholipids, explaining its superior activity. This structure-activity relationship guided our selection of **C17** for subsequent mechanistic studies. Notably, the MIC of **C17** against *S. aureus* ATCC 29213 (16 μg/mL) is higher than that of the clinical comparator vancomycin (1 μg/mL; Table 1). However, vancomycin is a last-resort glycopeptide antibiotic with known nephrotoxicity concerns and rising resistance rates (Mühlberg et al., 2020). In contrast, **C17** represents a novel chemical scaffold with a distinct membrane-targeting mechanism, which may offer advantages in combating vancomycin-intermediate *S. aureus* strains. Furthermore, the observed MIC values are comparable to those reported for other synthetic membrane-active hydrazide derivatives (MIC range 8–64 μg/mL against *S. aureus*) (Mbaveng et al., 2016; Xia et al., 2015), suggesting that the p-toluic hydrazide scaffold is a viable starting point for further optimization.

**TABLE 1:** MIC^a^ (μg/mL) of Schiff base derivatives.

Compounds	R	Ec	Se	Sa1	Sa2	MRSA2	Bs
**Van** ^b^	-^d^	-	-	1	1	1	1
**Enr** ^c^	-	0.0625	0.0625	-	-	-	-
**C1**	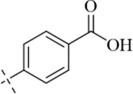	>256	>256	>256	>256	>256	>256
**C2**	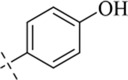	>256	>256	>256	>256	>256	>256
**C3**	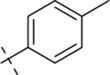	>256	>256	>256	>256	>256	>256
**C4**	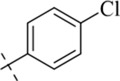	>256	>256	>256	>256	>256	>256
**C5**	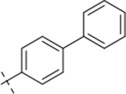	>256	>256	>256	>256	>256	>256
**C6**	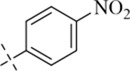	>256	>256	>256	>256	>256	>256
**C7**	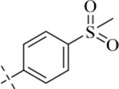	>256	>256	128	128	128	128
**C8**	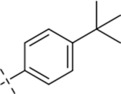	>256	>256	>256	>256	>256	>256
**C9**	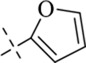	>256	>256	128	128	128	128
**C10**	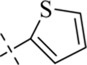	>256	>256	128	128	128	128
**C11**	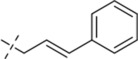	>256	>256	>256	>256	>256	>256
**C12**	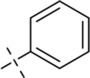	>256	>256	>256	>256	>256	>256
**C13**	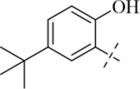	>256	>256	128	128	128	128
**C14**	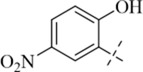	>256	>256	128	128	128	128
**C15**	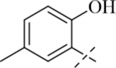	256	256	128	128	128	256
**C16**	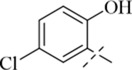	256	256	128	128	128	256
**C17**	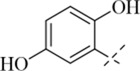	256	256	16	16	16	32
**C18**	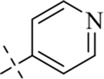	>256	>256	>256	>256	>256	>256

^a^ The minimum inhibitory concentration (MIC) is the lowest concentration that prevents visible microbial growth after 24 h. All experiments were performed in triplicate.

^b^ Van = vancomycin is a clinical drug against Gram-positive bacteria.

^c^ Enr = Enrofloxacin is a broad-spectrum quinolone-based antibiotic.

^d^ The dash “–” indicates not tested (for vancomycin and enrofloxacin).

Abbreviations: Ec = *Escherichia coli* ATCC, 25922; Se = *Salmonella enterica* serovar Enteritidis SM012; Sa1 = *Staphylococcus aureus* ATCC, 29213; Sa2 = *S. aureus* ATCC, 43300; MRSA2 = methicillin-resistant *S. aureus* MRSA2; Bs = *Bacillus subtilis* ATCC, 6633.

**TABLE 2:** The antibacterial activity of **C17**.

Compounds	*B. cereus* CMCC63303	*L. monocytogenes* CICC21662	*E*. *faecalis* ATCC29212	*S. aureus* LN38	*S. aureus* LN51
**Van**	2	2	2	2	2
**C17**	32	16	16	16	16

### 2.3 The toxicity of C17

**FIGURE 2:**
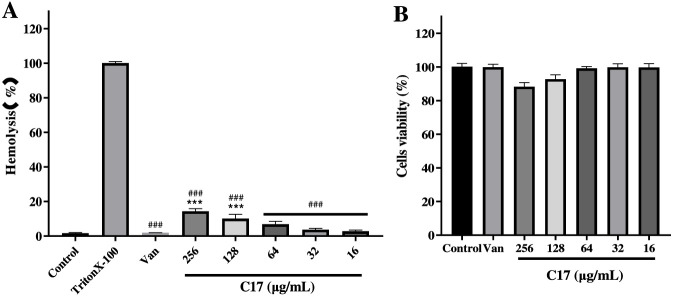
**(A)** Percentage of hemolysis of rabbit red blood cells at various **C17** concentrations. **(B)** Cytotoxicity of **C17** against Vero cells after 24 h. Untreated control (PBS for hemolysis; culture medium for cytotoxicity), positive control (1% Triton X-100 for hemolysis), and reference drug (Vancomycin 256 μg/mL for cytotoxicity) are indicated. Data are presented as means ± SEM from three independent experiments.*p < 0.05, **p < 0.01, ***p < 0.001 vs. untreated control; ^#^p < 0.05, ^##^p < 0.01, ^###^p < 0.001 vs. Triton X-100.

To comprehensively evaluate the biosafety of the active compound, hemolytic activity assays were first conducted for compound **C17** (Liu et al., 2024). As shown in Figure 2A, **C17** exhibited no visible hemolytic activity across the concentration range of 16–256 μg/mL, a profile comparable to the negative control (PBS) and in stark contrast to the positive control (1% Triton X-100) which induced complete hemolysis. Moreover, its effective antibacterial concentration is well below the hemolytic threshold, suggesting favorable hemocompatibility. Cytotoxicity was evaluated in VERO cells (Li et al., 2025). As presented in Figure 2B, **C17** did not induce any significant reduction in metabolic activity even at the highest concentration tested (256 μg/mL). To place these safety data in context, we calculated the selectivity index (SI = HC_50_/MIC) for **C17**. Using the highest tested non-hemolytic concentration (256 μg/mL) as a conservative estimate, the SI exceeds 16 (256/16) against *S. aureus*. This therapeutic window compares favorably with that of the membrane-active peptide melittin, which typically exhibits an SI < 5 due to pronounced hemolysis (Yang et al., 2024). Moreover, the absence of cytotoxicity in VERO cells at 256 μg/mL is consistent with previous reports on hydrazide-based Schiff bases, which generally display low mammalian toxicity owing to their selective interaction with bacterial membrane components (Reshma et al., 2022; Şenol et al., 2023). These findings indicate that **C17** exhibits a high level of safety toward mammalian cells under *in vitro* conditions, providing preliminary toxicological support for its further biological applications. Having confirmed the favorable safety profile of **C17**, we next evaluated its bactericidal kinetics and resistance propensity.

### 2.4 Time-killing curve determinations and drug resistance study

**FIGURE 3:**
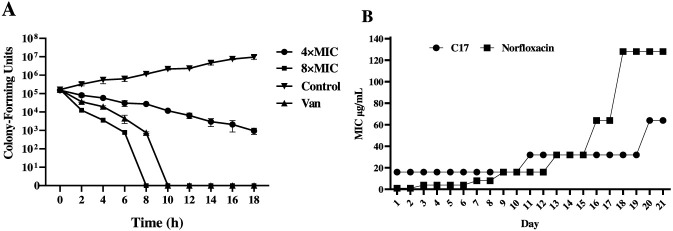
**(A)** Time-kill kinetics of **C17** against *S. aureus* ATCC 29213. **(B)** Resistance development of **C17** (fold change in MIC over 21 passages). Untreated control (DMSO only), Vancomycin (8 × MIC), and Norfloxacin (for resistance study) are shown as references. Data are presented as means ± SEM from three independent experiments.

To systematically evaluate the antibacterial properties of compound **C17**, this study focused on its bactericidal kinetics and potential for resistance induction against *S*. *aureus* ATCC 29213. As a common pathogen prone to developing resistance, *S*. *aureus* represents a critical model for assessing the efficacy and resistance control of new antibacterial agents (Li et al., 2025). At 8 × MIC, **C17** completely eradicated the bacterial culture within 4 h (Figure 3A), demonstrating rapid and concentration-dependent killing. This fast action is consistent with a membrane-disrupting mechanism, which typically acts more quickly than inhibitors of protein or nucleic acid synthesis. We next evaluated the propensity for resistance development by serial passaging. After 21 days of subculturing in sub-inhibitory concentrations of **C17**, the MIC increased less than 8-fold (Figure 3B), and the spontaneous resistance frequency remained low. This low resistance potential is likely attributable to the multi-target nature of membrane disruption. Thus, **C17** exhibits a high resistance barrier, a desirable feature for combating chronic biofilm infections. The rapid bactericidal kinetics of **C17** (complete eradication within 4 h at 8 × MIC) are characteristic of membrane-disrupting agents, which typically act faster than bacteriostatic drugs or inhibitors of macromolecular synthesis (Koh et al., 2016). For comparison, vancomycin requires 8–12 h to achieve a comparable 3-log reduction against *S. aureus* under similar conditions (Li et al., 2025). This rapid action may translate into faster resolution of infection and reduced opportunity for resistance emergence. Indeed, the low propensity for resistance development (<8-fold MIC increase over 21 passages) is consistent with the multi-target nature of membrane disruption, which imposes a high evolutionary barrier. Similar resistance-suppressing properties have been documented for other membrane-active compounds, including daptomycin and certain antimicrobial peptides (Etayash et al., 2020). The rapid bactericidal effect and low resistance frequency prompted us to investigate the underlying mechanism, focusing on the bacterial membrane as a potential target.

### 2.5 Antimicrobial mechanism investigation

**FIGURE 4:**
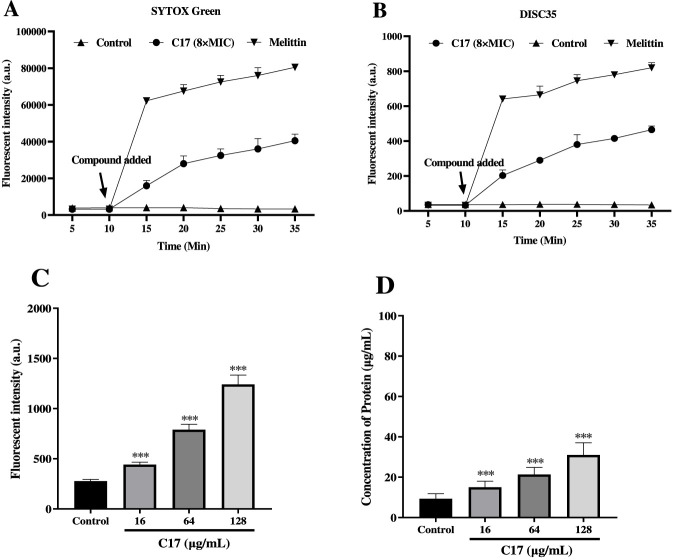
**(A)** Cytoplasmic membrane permeabilization by **C17** assessed using SYTOX Green uptake. **(B)** Cytoplasmic membrane depolarization by **C17** measured with the DiSC_35_ probe. **(C)** Effect of **C17** treatment on reactive oxygen species (ROS) production in *S. aureus* ATCC 29213. **(D)** Protein leakage caused by **C17** in *S. aureus* ATCC 29213. Untreated control (bacteria without compound) and positive control (Melittin, 8 × MIC) are indicated. *p < 0.05, **p < 0.01, ***p < 0.001 vs. untreated control. Data are presented as means ± SEM from three independent experiments.

To test whether **C17** acts on the bacterial membrane, we first monitored changes in membrane potential and permeability in real time (Koh et al., 2016). Within 10 min of adding **C17** (8 × MIC) to *S. aureus* ATCC 29213 suspensions, the fluorescence intensity of the membrane potential-sensitive dye DiSC_35_ increased continuously (Figure 4B), indicating rapid membrane depolarization. Concurrently, the membrane-impermeable dye SYTOX Green showed a marked fluorescence increase (Figure 4A), demonstrating loss of membrane integrity and increased permeability. These changes occurred within the first 35 min and were concentration-dependent. In contrast, no fluorescence increase was observed in the untreated control, confirming that the effects are specifically caused by **C17**. These results establish that **C17** disrupts bacterial membrane polarization and barrier function.

Having established that **C17** disrupts membrane integrity, we next asked whether this leads to downstream oxidative stress. Disruption of membrane homeostasis often induces accumulation of reactive oxygen species (ROS), a common bactericidal mechanism (Yang et al., 2024). As shown in Figure 4C, within 30 min of exposure to **C17**, a significant increase in ROS levels was observed, indicating activation of oxidative stress. This change showed a clear correlation with increased bacterial mortality, suggesting that ROS accumulation likely plays an important role in the bactericidal mechanism of **C17**.

Finally, to directly demonstrate loss of membrane barrier function, we quantified protein leakage into the culture medium. As illustrated in Figure 4D, compared with the blank control group, the extracellular protein concentration increased significantly in the **C17**-treated bacterial suspensions, exhibiting a clear dose-dependent manner. This result provides direct evidence that **C17** disrupts the structural integrity of the bacterial cell membrane, leading to the release of biological macromolecules such as proteins, and ultimately resulting in bacterial cell death. Collectively, these data support that **C17** acts primarily as a membrane-active antibacterial agent.

These data establish **C17** as a membrane-active antibacterial agent with a clear temporal sequence: membrane depolarization and permeabilization within 10–35 min, followed by ROS accumulation within 30 min and protein leakage thereafter. The rapid onset distinguishes **C17** from slower-acting cell wall or protein synthesis inhibitors such as vancomycin and linezolid (Koh et al., 2016; Li et al., 2025). The delayed ROS accumulation indicates that oxidative stress is a consequence, not a cause, of the primary membrane damage, consistent with reports that membrane permeabilization triggers secondary stress responses (Yang et al., 2024). The protein leakage profile aligns with that of membrane-active peptides like melittin (Etayash et al., 2020); however, **C17** exhibits superior selectivity, with minimal hemolysis (Figure 2A), likely due to the 2,5-dihydroxy substitution pattern. This multi-target membrane-disrupting mechanism explains both the rapid killing kinetics (Figure 3A) and the low resistance propensity (Figure 3B), as simultaneous mutations in multiple membrane components are statistically improbable. Thus, **C17** operates via a mechanism distinct from conventional single-target antibiotics, supporting its potential against drug-resistant Gram-positive pathogens.

### 2.6 The anti-inflammatory activity of C17

**FIGURE 5:**
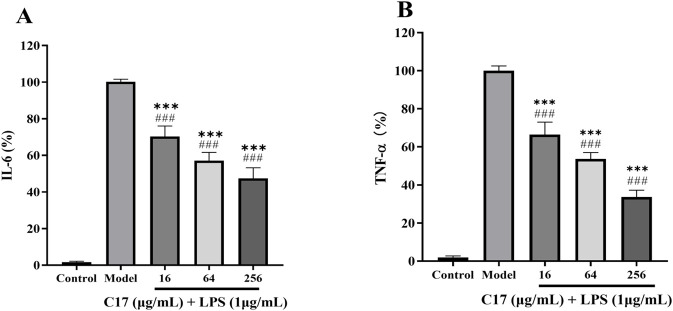
Anti-inflammatory activity of **C17** in LPS-stimulated RAW 264.7 macrophages. **(A)** IL-6 levels. **(B)** TNF-α levels. Untreated control (cells without LPS), LPS model group (LPS only), and LPS + **C17** groups are shown. *p < 0.05, **p < 0.01, ***p < 0.001 vs. LPS model group; ^###^p < 0.001 vs. untreated control. Data are presented as means ± SEM from three independent experiments.

Given the literature precedent for anti-inflammatory activity of Schiff bases (Liu et al., 2018; Mbaveng et al., 2016), we examined whether **C17** modulates cytokine production in LPS-stimulated macrophages. As shown in Figure 5, LPS markedly increased the secretion of IL-6 and TNF-α. Co-treatment with **C17** significantly suppressed both cytokines in a concentration-dependent manner, with effective inhibition observed at 64 μg/mL (4 × MIC). Notably, this concentration was previously confirmed to be non-cytotoxic to RAW 264.7 macrophages (data not shown), indicating that the observed suppression is not an artefact of reduced cell viability. The anti-inflammatory effect of **C17** may complement its direct antibacterial activity. By reducing the production of pro-inflammatory cytokines such as IL-6 and TNF-α, **C17** could potentially mitigate excessive inflammation that often accompanies severe bacterial infections, thereby limiting tissue damage while eradicating the pathogen. This dual action is particularly advantageous for treating biofilm-associated chronic infections where persistent inflammation impairs healing.

While the present data demonstrate clear anti-inflammatory potential for **C17**, the underlying mechanism remains to be elucidated. One plausible pathway is the NF-κB signaling cascade, a common target in LPS-stimulated macrophages. Future studies using Western blotting or immunofluorescence are needed to verify this. We also acknowledge that only two pro-inflammatory cytokines (IL-6 and TNF-α) were examined; a broader panel (e.g., IL-1β, IL-10) would provide a more complete immunomodulatory profile. **C17** was selected for testing based on its superior antibacterial and antibiofilm activities; whether other analogues exhibit similar effects awaits future structure-activity relationship studies. Nevertheless, the dual antibacterial and anti-inflammatory profile of **C17** distinguishes it from conventional antibiotics such as vancomycin, which lack intrinsic immunomodulatory activity. Schiff base derivatives have been previously reported to inhibit NF-κB activation (Wang S. et al., 2023), and the potency of **C17** (significant suppression at 64 μg/mL, corresponding to 4 × MIC) is comparable to that of curcumin, a well-characterized natural anti-inflammatory agent, in similar LPS-stimulated macrophage models (Xiao et al., 2025). This dual functionality is particularly relevant for biofilm-associated infections, where persistent inflammation contributes to tissue destruction and impaired healing. Despite these limitations, the combined antibacterial and anti-inflammatory actions of **C17** support its therapeutic potential for chronic biofilm-associated infections, where conventional antibiotics often fail due to unresolved inflammation.

### 2.7 Inhibitory effects towards *S. aureus* biofilm formation

**FIGURE 6:**
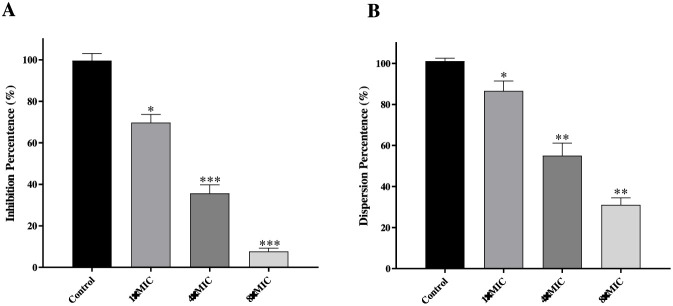
**(A)** Inhibition of *S. aureus* ATCC 29213 biofilm formation by **C17**. **(B)** Eradication of pre-formed *S. aureus* ATCC 29213 biofilms by **C17**. Untreated control (bacteria without compound) is shown. *p < 0.05, **p < 0.01, ***p < 0.001 vs. untreated control. Data are presented as means ± SD from three independent experiments.

Over 80% of chronic bacterial infections in humans are linked to biofilms. These structured microbial communities are embedded in a protective extracellular matrix that greatly increases their resistance to antibacterial drugs and host immunity. Typical biofilm-related infections occur on medical devices such as catheters and implants, in chronic wounds, and in the respiratory tract of cystic fibrosis patients, where they often lead to persistent, recurrent, and difficult-to-treat conditions. Hence, developing agents that can prevent biofilm formation and eliminate mature biofilms is critically needed (Etayash et al., 2020; Yang et al., 2024). In this context, the activity of compound **C17** against *S*. *aureus* ATCC 29213 biofilms was examined. Using crystal violet staining, biofilm formation was quantified, showing concentration-dependent inhibition (Figure 6A): at 16 μg/mL (1×MIC), 64 μg/mL (4×MIC), and 256 μg/mL (8×MIC), inhibition rates were 30%, 64%, and 92%, respectively. The efficacy of **C17** in disrupting preformed biofilms was also assessed (Figure 6B). Removal rates reached 13%, 45%, and 69% at the same concentrations, demonstrating its potential to eradicate established *S. aureus* biofilms. The biofilm inhibitory activity of **C17** (92% inhibition at 8 × MIC) is comparable to that reported for the synthetic polymer Bu:DM, which achieves approximately 80% reduction in viability at similar multiples of MIC (Etayash et al., 2020). Notably, **C17** also eradicated pre-formed biofilms by 69% at 8 × MIC, a challenging feat given the intrinsic tolerance of biofilm-embedded cells to antimicrobials. This biofilm-eradicating capacity is likely attributable to the membrane-disrupting mechanism, which does not require active bacterial metabolism and is therefore effective against dormant persister cells within the biofilm matrix (Yang et al., 2024). In contrast, vancomycin exhibits poor biofilm penetration and negligible activity against biofilm-embedded *S. aureus* unless combined with rifampicin. Thus, **C17** offers a distinct advantage in treating chronic biofilm-associated infections.

### 2.8 Evaluation of the drug-likeness of C17

To systematically evaluate the development potential of the synthesized Schiff base derivative **C17** as a drug candidate, key druggability studies were conducted following the demonstration of its significant anti-Gram-positive bacterial activity, low systemic toxicity, unique antibacterial mechanism, and biofilm eradication capacity. These studies aimed to comprehensively predict its *in vivo* pharmacokinetic behavior and development prospects. As summarized in Table 3, **C17** exhibited a high human plasma protein binding (PPB) rate of 89.3%, indicating extensive binding to blood proteins that may significantly limit the free drug concentration. This characteristic should be carefully considered in subsequent dose design to achieve effective systemic exposure and desired antibacterial effects. Given the MIC of **C17** against *S. aureus* is 16 μg/mL and the PPB is 89.3%, the free fraction is only 10.7%. Consequently, the total plasma concentration required to reach the MIC would be approximately 150 μg/mL. This high requirement poses a major hurdle for *in vivo* efficacy, especially considering the moderate lipophilicity (logD_7_._4_ = 3.14) which may limit solubility. Thus, reducing PPB is a key goal for future structural optimization. For context, the free fraction of **C17** (10.7%) is lower than that of most oral antibiotics (e.g., ciprofloxacin ∼70% unbound, linezolid ∼69% unbound) but falls within the range observed for highly lipophilic anti-infectives such as azithromycin (PPB ∼85–93%). Given that **C17** is envisioned for topical or local administration in biofilm-associated infections, the impact of high PPB on systemic exposure may be mitigated. The n-octanol/water partition coefficient (logD_7.4_) was determined to be 3.14, reflecting moderate lipophilicity at physiological pH. This property is likely beneficial for penetrating bacterial cell membranes and biofilm structures, thereby enhancing antibacterial efficacy; however, it may also imply limited aqueous solubility, highlighting the need for optimization of solubility and bioavailability during formulation development. In metabolic stability studies, **C17** showed a half-life (T_1_/_2_) of 52.98 min and a total clearance (CL) of 9.12 μL/min/mg in liver microsomes, indicating intermediate metabolic stability and a relatively low hepatic extraction ratio. Although not as stable as some highly optimized lead compounds, these values suggest acceptable *in vivo* residence time and potential sustained antibacterial activity, providing a foundation for further pharmacokinetic/pharmacodynamic studies. In conclusion, **C17** demonstrates several promising druggability advantages, including suitable lipophilicity and acceptable metabolic stability. However, challenges such as high plasma protein binding and potentially limited solubility were also identified. These findings provide clear guidance for future structural optimization and formulation strategies, underscoring the compound’s promise as a antibacterial lead candidate.

**TABLE 3:** Partial drug likeness data for **C17**.

Compound	PPB	logD_7.4_	T_1/2_ (min)	CL (μL/min/mg)
**C17**	89.3%	3.14 ± 0.12	52.98	9.12

## 3 Conclusion

This study developed a novel Schiff base derivative, **C17**, which exhibits potent antibacterial activity against Gram-positive pathogens—particularly *S*. *aureus*—with a MIC value of 16 μg/mL. **C17** demonstrated a favorable safety profile, including no hemolytic activity and minimal cytotoxicity toward mammalian cells. Its rapid and concentration-dependent bactericidal effects were associated with membrane disruption, evidenced by membrane depolarization, increased permeability, ROS generation, and protein leakage. Furthermore, **C17** showed a low tendency to induce resistance and exhibited effective anti-biofilm activity, inhibiting biofilm formation as well as eradicating mature biofilms. Although high plasma protein binding may limit free drug concentration, its moderate lipophilicity and metabolic stability support its potential for further development. Overall, **C17** represents a promising multifunctional antibacterial agent with potential to overcome drug resistance and treat biofilm-associated infections.

## 4 Experimental section

### 4.1 Chemically synthetical experiments

All commercial chemicals were used as received from Adamas. Solvents were employed without further purification or dried over molecular sieves when necessary. TLC analysis was performed on GF254 plates. NMR spectra (^1^H: 400 MHz; ^13^C: 100 MHz) were recorded on a Bruker Avance 400 spectrometer, and are referenced to residual solvent peaks (DMSO: δH 2.5 ppm, δC 39.5 ppm). High-resolution MS (HRMS) was obtained using an AB Sciex TripleTOF 5600+ instrument with ESI ionization.

#### 4.1.1 4-{[2-(4-methylbenzoyl)hydrazineylidene]methyl}benzoic acid (C1)

4-Methylbenzohydrazide (1 mmol) was dissolved in anhydrous ethanol. Subsequently, an aldehyde derivative (1.05 mmol) was added, and the solution was heated under reflux at 80 °C for 8 h. Upon completion of the reaction, the final product **C** was obtained by recrystallization from ethanol.

Compound **C1** was synthesized analogously to compound **B**. Compound **B** (1 mmol) was dissolved in anhydrous ethanol, followed by the addition of 4-chloro-3-hydroxybenzaldehyde (1 mmol). The solution was heated to reflux at 80 °C for 8 h. Upon reaction completion, the product was obtained by recrystallization from ethanol.

239 mg, Yield, 85%. White solid powder. M.P. 257 °C–259 °C. ^1^H NMR (400 MHz, DMSO-*d*_6_) δ 11.93 (s, 1H), 8.51 (s, 1H), 8.01 (d, *J* = 7.9 Hz, 2H), 7.84 (d, *J* = 6.6 Hz, 4H), 7.33 (d, *J* = 7.9 Hz, 2H), 2.37 (s, 3H). ^13^C NMR (100 MHz, DMSO-*d*_6_) δ 166.90, 163.12, 146.29, 141.96, 138.44, 131.66, 129.77, 129.00, 127.70, 127.02, 21.02. TOF-MS, m/z: [M + H]^+^, calcd. for C_16_H_15_N_2_O_3_^+^, 283.1082, found: 283.1085.

#### 4.1.2 *N*′-(4-hydroxybenzylidene)-4-methylbenzohydrazide (C2)

208 mg, Yield, 82%. White solid powder. M.P. 193 °C–195 °C. ^1^H NMR (400 MHz, DMSO-*d*_6_) δ 11.58 (s, 1H), 9.92 (s, 1H), 8.36 (s, 1H), 7.82 (d, *J* = 7.8 Hz, 2H), 7.56 (d, *J* = 8.3 Hz, 2H), 7.31 (d, *J* = 7.9 Hz, 2H), 6.85 (d, *J* = 8.3 Hz, 2H), 2.37 (s, 3H). ^13^C NMR (100 MHz, DMSO-*d*_6_) δ 162.71, 159.36, 147.89, 141.53, 130.76, 128.91, 128.78, 127.52, 125.38, 115.69, 20.98. TOF-MS, m/z: [M + H]^+^, calcd. for C_15_H_15_N_2_O_2_^+^, 255.1133, found: 255.1137.

#### 4.1.3 4-methyl-*N*′-(4-methylbenzylidene)benzohydrazide (C3)

231 mg, Yield, 92%. White solid powder. M.P. 176 °C–178 °C. ^1^H NMR (400 MHz, DMSO-*d*_6_) δ 11.73 (s, 1H), 8.43 (s, 1H), 7.84 (d, *J* = 7.7 Hz, 2H), 7.62 (d, *J* = 7.6 Hz, 2H), 7.29 (dd, *J* = 24.2, 7.8 Hz, 4H), 2.35 (d, *J* = 14.4 Hz, 6H). ^13^C NMR (100 MHz, DMSO-*d*_6_) δ 163.10, 147.79, 141.89, 139.99, 131.90, 129.61, 129.15, 127.79, 127.21, 21.19. TOF-MS, m/z: [M + H]^+^, calcd. for C_16_H_17_N_2_O^+^, 253.1341, found: 253.1345.

#### 4.1.4 *N*′-(4-chlorobenzylidene)-4-methylbenzohydrazide (C4)

220 mg, Yield, 81%. White solid powder. M.P. 169 °C–171 °C. ^1^H NMR (400 MHz, DMSO-d6) δ 11.82 (s, 1H), 8.51 (s, 1H), 7.84 (m, 4H), 7.55–7.26 (m, 4H), 2.39 (s, 3H). ^13^C NMR (100 MHz, DMSO-*d*_6_) δ 163.12, 147.25, 141.97, 141.67, 139.52, 139.70, 129.18, 128.02, 127.81, 127.22, 126.83, 21.20. TOF-MS, m/z: [M + H]^+^, calcd. for C_15_H_14_ClN_2_O^+^, 273.0794, found: 273.0796.

#### 4.1.5 *N*′-[(1,1′-biphenyl)-4-ylmethylene]-4-methylbenzohydrazide (C5)

283 mg, Yield, 90%. White solid powder. M.P. 184 °C–186 °C. ^1^H NMR (400 MHz, DMSO-*d*_6_) δ 11.85 (s, 1H), 8.45 (s, 1H), 7.79 (dd, *J* = 37.4, 7.7 Hz, 4H), 7.41 (dd, *J* = 73.0, 7.9 Hz, 4H), 2.37 (s, 3H). ^13^C NMR (100 MHz, DMSO-*d*_6_) δ 162.80, 145.95, 141.63, 134.21, 133.13, 130.22, 128.75, 128.67, 128.41, 127.42, 20.78. TOF-MS, m/z: [M + H]^+^, calcd. for C_21_H_19_N_2_O^+^, 315.1497, found: 315.1499.

#### 4.1.6 *N*′-(2-hydroxy-5-nitrobenzylidene)isonicotinohydrazide (C6)

258 mg, Yield, 91%. White solid powder. M.P. 183 °C–185 °C. ^1^H NMR (400 MHz, DMSO-*d*_6_) δ 12.05 (s, 1H), 8.53 (s, 1H), 8.27 (d, *J* = 8.1 Hz, 2H), 7.89 (dd, *J* = 53.5, 6.6 Hz, 4H), 7.32 (d, *J* = 7.4 Hz, 2H), 2.36 (s, 3H). ^13^C NMR (100 MHz, DMSO-*d*_6_) δ 162.98, 147.56, 144.72, 141.90, 140.50, 129.96, 128.79, 127.68, 127.53, 123.81, 20.80. TOF-MS, m/z: [M + H]^+^, calcd. for C_15_H_14_N_3_O_3_^+^, 284.1035, found: 284.1038.

#### 4.1.7 4-methyl-*N*′-[4-(methylsulfonyl)benzylidene]benzohydrazide (C7)

269 mg, Yield, 85%. White solid powder. M.P. 157 °C–159 °C. ^1^H NMR (400 MHz, DMSO-*d*_6_) δ 12.02 (s, 1H), 8.54 (s, 1H), 8.09–7.79 (m, 5H), 7.35 (d, *J* = 7.9 Hz, 2H), 3.26 (s, 3H), 2.39 (s, 3H).^13^C NMR (100 MHz, DMSO-*d*_6_) δ 162.97, 145.29, 141.84, 141.13, 138.99, 130.04, 128.80, 127.40, 127.28, 43.23, 20.81. TOF-MS, m/z: [M + H]^+^, calcd. for C_16_H_17_N_2_O_3_S^+^, 317.0960, found: 317.0962.

#### 4.1.8 *N*′-[4-(tert-butyl)benzylidene]-4-methylbenzohydrazide (C8)

262 mg, Yield, 89%. White solid powder. M.P. 152 °C–154 °C. ^1^H NMR (400 MHz, DMSO-*d*_6_) δ 11.70 (s, 1H), 8.42 (s, 1H), 7.81 (d, *J* = 7.7 Hz, 2H), 7.63 (d, *J* = 7.9 Hz, 2H), 7.46 (d, *J* = 8.0 Hz, 2H), 7.30 (d, *J* = 7.8 Hz, 2H), 2.35 (s, 3H), 1.27 (s, 9H). ^13^C NMR (100 MHz, DMSO-*d*_6_) δ 162.86, 152.78, 147.50, 141.68, 131.67, 130.59, 128.94, 127.57, 126.85, 125.59, 34.55, 30.93, 20.99. TOF-MS, m/z: [M + H]^+^, calcd. for C_19_H_23_N_2_O^+^, 295.1810, found: 295.1813.

#### 4.1.9 *N*′-(furan-2-ylmethylene)-4-methylbenzohydrazide (C9)

189 mg, Yield, 83%. White solid powder. M.P. 210 °C–212 °C. ^1^H NMR (400 MHz, DMSO-*d*_6_) δ 11.73 (s, 1H), 8.36 (s, 1H), 7.83 (d, *J* = 6.7 Hz, 3H), 7.32 (d, *J* = 7.8 Hz, 2H), 6.91 (s, 1H), 6.63 (s, 1H), 2.37 (s, 3H). ^13^C NMR (100 MHz, DMSO-*d*_6_) δ 162.90, 149.52, 145.06, 141.79, 137.33, 130.46, 128.98, 127.57, 113.24, 112.15, 20.99. TOF-MS, m/z: [M + H]^+^, calcd. for C_13_H_13_N_2_O_2_^+^, 229.0977, found: 229.0979.

#### 4.1.10 4-methyl-*N*′-(thiophen-2-ylmethylene)benzohydrazide (C10)

198 mg, Yield, 81%. White solid powder. M.P. 183 °C–185 °C. ^1^H NMR (400 MHz, DMSO-*d*_6_) δ 11.77 (s, 1H), 8.69 (s, 1H), 7.83 (d, *J* = 7.8 Hz, 2H), 7.65 (d, *J* = 4.6 Hz, 1H), 7.45 (s, 1H), 7.31 (d, *J* = 7.8 Hz, 2H), 7.13 (d, *J* = 4.2 Hz, 1H), 2.36 (s, 3H). ^13^C NMR (100 MHz, DMSO-*d*_6_) δ 163.10, 142.92, 141.99, 139.43, 130.95, 130.72, 129.19, 129.02, 128.03, 127.79, 21.21. TOF-MS, m/z: [M + H]^+^, calcd. for C_13_H_13_N_3_OS^+^, 245.1748, found: 245.1751.

#### 4.1.11 4-methyl-*N*′-(4-phenylbut-3-en-1-ylidene)benzohydrazide (C11)

245 mg, Yield, 88%. White solid powder. M.P. 158 °C–160 °C. ^1^H NMR (400 MHz, DMSO-*d*_6_) δ 11.67 (s, 1H), 8.25 (d, *J* = 7.1 Hz, 1H), 7.72 (dd, *J* = 79.5, 7.5 Hz, 4H), 7.46–7.23 (m, 5H), 7.05 (s, 2H), 2.37 (s, 3H). ^13^C NMR (100 MHz, DMSO-*d*_6_) δ 162.79, 149.45, 141.71, 138.77, 135.92, 130.51, 128.93, 128.78, 127.60, 127.03, 125.72, 20.99. TOF-MS, m/z: [M + H]^+^, calcd. for C_18_H_19_N_2_O^+^, 279.1497, found: 279.1499.

#### 4.1.12 *N′-*benzylidene-4-methylbenzohydrazide (C12)

212 mg, Yield, 89%. White solid powder. M.P. 165 °C–167 °C. ^1^H NMR (400 MHz, DMSO-*d*_6_) δ 11.76 (s, 1H), 8.45 (s, 1H), 7.90–7.59 (m, 4H), 7.58–7.18 (m, 4H), 2.36 (s, 3H). ^13^C NMR (100 MHz, DMSO-*d*_6_) δ 163.15, 147.71, 141.97, 134.59, 130.17, 129.17, 129.01, 127.81, 127.21, 21.20. TOF-MS, m/z: [M + H]^+^, calcd. for C_15_H_15_N_2_O^+^, 239.1184, found: 239.1187.

#### 4.1.13 *N*′-[5-(tert-butyl)-2-hydroxybenzylidene]-4-methylbenzohydrazide (C13)

264 mg, Yield, 85%. White solid powder. M.P. 163 °C–165 °C. ^1^H NMR (400 MHz, DMSO-*d*_6_) δ 12.00 (s, 1H), 11.12 (s, 1H), 8.62 (s, 1H), 7.83 (d, *J* = 7.9 Hz, 2H), 7.47 (s, 1H), 7.32 (d, *J* = 7.7 Hz, 3H), 6.85 (d, *J* = 8.6 Hz, 1H), 2.36 (s, 3H), 1.25 (s, 9H). ^13^C NMR (100 MHz, DMSO-*d*_6_) δ 162.88, 155.51, 148.77, 142.21, 141.64, 130.20, 129.24, 128.67, 127.84, 125.86, 118.11, 116.24, 39.94, 31.42, 21.22. TOF-MS, m/z: [M + H]^+^, calcd. for C_19_H_23_N_2_O_2_^+^, 311.1759, found: 311.1762.

#### 4.1.14 *N*′-(2-hydroxy-5-nitrobenzylidene)-4-methylbenzohydrazide (C14)

251 mg, Yield, 84%. White solid powder. M.P. 193 °C–195 °C. ^1^H NMR (400 MHz, DMSO-*d*_6_) δ 8.73 (s, 1H), 8.58 (s, 1H), 8.17 (d, *J* = 8.9 Hz, 1H), 7.86 (d, *J* = 6.7 Hz, 2H), 7.35 (d, *J* = 7.0 Hz, 2H), 7.11 (d, *J* = 8.9 Hz, 1H), 2.39 (s, 3H). ^13^C NMR (100 MHz, DMSO-*d*_6_) δ 162.38, 144.01, 142.01, 139.70, 129.55, 128.85, 127.51, 126.25, 123.66, 119.75, 116.91, 20.82. TOF-MS, m/z: [M + H]^+^, calcd. for C_15_H_14_N_3_O_4_^+^, 300.0984, found: 300.0987.

#### 4.1.15 *N*′-(2-hydroxy-5-methylbenzylidene)-4-methylbenzohydrazide (C15)

228 mg, Yield, 85%. White solid powder. M.P. 204 °C–206 °C. ^1^H NMR (400 MHz, DMSO-*d*_6_) δ 12.02 (s, 1H), 11.08 (s, 1H), 8.59 (s, 1H), 7.85 (d, *J* = 7.9 Hz, 2H), 7.34 (d, *J* = 8.5 Hz, 3H), 7.10 (d, *J* = 8.1 Hz, 1H), 6.83 (d, *J* = 8.3 Hz, 1H), 2.38 (s, 3H), 2.25 (s, 3H). ^13^C NMR (100 MHz, DMSO-*d*_6_) δ 162.82, 155.51, 148.28, 142.23, 132.17, 130.14, 129.61, 129.24, 128.06, 127.84, 118.54, 116.45, 21.22, 20.12. TOF-MS, m/z: [M + H]^+^, calcd. for C_16_H_17_N_2_O_2_^+^, 269.1290, found: 269.1293.

#### 4.1.16 *N*′-(5-chloro-2-hydroxybenzylidene)-4-methylbenzohydrazide (C16)

230 mg, Yield, 80%. White solid powder. M.P. 175 °C–177 °C. ^1^H NMR (400 MHz, DMSO-*d*_6_) δ 12.13 (s, 1H), 11.37 (s, 1H), 8.62 (s, 1H), 7.86 (d, *J* = 7.9 Hz, 2H), 7.64 (s, 1H), 7.38–7.18 (m, 3H), 6.95 (d, *J* = 8.8 Hz, 1H), 2.36 (s, 3H). ^13^C NMR (100 MHz, DMSO-*d*_6_) δ 162.81, 156.08, 145.84, 142.13, 130.63, 129.82, 129.03, 127.80, 127.70, 122.96, 120.64, 118.19, 21.02. TOF-MS, m/z: [M + H]^+^, calcd. for C_15_H_14_ClN_2_O_2_^+^, 289.0744, found: 289.0747.

#### 4.1.17 *N*′-(2,5-dihydroxybenzylidene)-4-methylbenzohydrazide (C17)

232 mg, Yield, 86%. White solid powder. M.P. 195 °C–197 °C. ^1^H NMR (400 MHz, DMSO-*d*_6_) δ 11.93 (s, 1H), 10.45 (s, 1H), 8.98 (s, 1H), 8.57 (s, 1H), 7.85 (d, *J* = 7.8 Hz, 2H), 7.33 (d, *J* = 7.9 Hz, 2H), 6.96 (s, 1H), 6.75 (s, 2H), 2.38 (s, 3H). ^13^C NMR (100 MHz, DMSO-*d*_6_) δ 162.42, 150.04, 149.66, 147.38, 141.78, 129.86, 128.84, 127.42, 118.79, 116.85, 113.79, 20.82. TOF-MS, m/z: [M + H]^+^, calcd. for C_15_H_15_N_2_O_3_^+^, 271.1082, found: 271.1085.

#### 4.1.18 4-methyl-*N*′-(pyridin-4-ylmethylene)benzohydrazide (C18)

203 mg, Yield, 85%. White solid powder. M.P. 191 °C–193 °C. ^1^H NMR (400 MHz, DMSO-*d*_6_) δ 12.05 (s, 1H), 8.64 (s, 2H), 8.45 (s, 1H), 7.85 (d, *J* = 7.2 Hz, 2H), 7.66 (s, 2H), 7.33 (d, *J* = 7.9 Hz, 2H), 2.37 (s, 3H). ^13^C NMR (100 MHz, DMSO-*d*_6_) δ 163.06, 150.04, 144.84, 141.36, 129.98, 128.83, 127.56, 120.75, 20.82. TOF-MS, m/z: [M + H]^+^, calcd. for C_14_H_14_N_3_O^+^, 240.1137, found: 240.1139.

### 4.2 Determination of minimum inhibitory concentration

The minimum inhibitory concentrations (MICs) of the synthesized compounds were assessed employing the broth microdilution technique in compliance with the guidelines established by the Clinical and Laboratory Standards Institute (CLSI) (Maia et al., 2022; Xia et al., 2015). Initially, microbial suspensions were prepared by culturing bacterial strains in Mueller-Hinton broth (MHB) at 37 °C under constant agitation until mid-logarithmic phase was reached. The bacterial inocula were then adjusted to an approximate density of 1 × 10^5^ colony-forming units per milliliter (CFU/mL). These adjusted suspensions were subsequently aliquoted into sterile 96-well microtiter plates. Test compounds, serially diluted in two-fold increments across a concentration range of 0.05–256 μg/mL, were introduced into the respective wells. Following an incubation period of 18 h at 37 °C, the MIC values were determined as the lowest compound concentration that completely inhibited visible bacterial growth. To ensure reliability and reproducibility, each assay was conducted in triplicate on independent occasions.

### 4.3 Time-killing kinetics

The time-kill kinetics of compound **C17** against *S. aureus* ATCC 29213 were evaluated using the viable plate count method (Li et al., 2025). Briefly, an overnight culture was diluted in fresh Mueller-Hinton broth to approximately 1 × 10^6^ CFU/mL and exposed to compound **C17** at concentrations corresponding to 4 × and 8 × the MIC. Aliquots were withdrawn at predetermined time intervals, serially diluted in sterile saline, and plated onto Mueller-Hinton agar plates. After incubation at 37 °C for 18–24 h, the number of viable colonies was enumerated. The limit of detection was 100 CFU/mL. Time-kill curves were generated by plotting log_10_ CFU/mL versus time. All experiments were performed in biological triplicate.

### 4.4 Drug resistance study

The potential for resistance development in *S. aureus* ATCC 29213 in response to compound **C17** was investigated using a multi-step serial passaging method, as previously described with minor modifications (Li et al., 2025). Briefly, the initial MIC of **C17** against the bacterium was determined using the broth microdilution method in accordance with CLSI guidelines. The strain was then subjected to repeated subculturing in Mueller-Hinton broth containing gradually increasing concentrations of **C17** over a period of 21 days. Daily, bacterial cultures were exposed to sub-inhibitory concentrations of the compound (0.5× to 2× MIC) and passaged into fresh medium containing the same or a higher concentration of **C17**. A ≥ 8-fold increase in MIC compared to the baseline was considered indicative of resistance development.

### 4.5 Hemolysis assay

The hemolytic activity of compound **C17** was evaluated according to a previously reported protocol with minor modifications (Li et al., 2025). Briefly, freshly prepared rabbit erythrocytes were washed and resuspended in phosphate-buffered saline (PBS) to obtain a 4% (v/v) suspension. Aliquots (100 µL) of this suspension were mixed with an equal volume of **C17** solution at various concentrations (ranging from 16 to 256 μg/mL) in PBS. Controls consisted of 1% Triton X-100 (for 100% hemolysis) and PBS (for background hemolysis). The mixtures were incubated at 37 °C for 1 h and then centrifuged at 1,000 *g* for 5 min. The release of hemoglobin was quantified by measuring the absorbance of the supernatant at 490 nm. The percentage of hemolysis was calculated using the following formula: Hemolysis (%) = [(Abs_sample_ − Abs_PBS_)/(Abs_Triton_ − Abs_PBS_)] × 100. All assays were conducted in triplicate to ensure reproducibility.

### 4.6 Cytotoxicity assay

The cytotoxicity of the test compounds was evaluated using the Cell Counting Kit-8, according to the manufacturer’s instructions with slight adaptations (Koh et al., 2016). Briefly, cells were seeded into 96-well plates at an appropriate density and allowed to adhere overnight. Subsequently, the cells were treated with various concentrations of the compounds for a specified period. After treatment, CCK-8 solution was added to each well, followed by incubation for an additional 1–4 h at 37 °C. The absorbance of each well was measured at 450 nm using a microplate reader. Cell viability was calculated based on the following equation: Cell viability (%) = [(OD_450,sample_ - OD_450,blank_)/(OD_450,control_ - OD_450,blank_)] × 100. All experiments were repeated three times.

### 4.7 Biofilm formation assay

The inhibitory effect of compound **C17** on biofilm formation was assessed using *S. aureus* ATCC 29213. Bacteria were diluted 100-fold in fresh tryptic soy broth (TSB) supplemented with 1% (w/v) glucose. Aliquots of the diluted culture were combined with varying concentrations of **C17** in 200 µL of the same medium per well. An equivalent volume of DMSO served as the negative control (Etayash et al., 2020; Yang et al., 2024). After 24 h of incubation at 37 °C, the formed biofilms were gently washed three times with PBS, air-dried, and stained with 0.1% crystal violet for 15 min. Excess stain was removed by repeated PBS washing, and the bound dye was solubilized in 95% ethanol. The absorbance was measured at 595 nm. The percentage of biofilm inhibition was determined using the formula: Inhibition (%) = [(OD_595_, control − OD_595_, sample)/OD_595_, control] × 100.

For the biofilm eradication assay, pre-formed biofilms were established by incubating bacterial suspensions for 24 h under identical conditions. Subsequently, the medium was carefully removed, and fresh TSB containing 1% glucose and sub-inhibitory concentrations of **C17** was added. The plates were incubated again at 37 °C for 24 h. The remaining steps—washing, staining, destaining, and quantification—were performed as described above. All assays were conducted in triplicate.

### 4.8 The anti-inflammatory activity of C17

This study evaluated the effects of the test compound **C17** on the production of inflammatory cytokines IL-6 and TNF-α in lipopolysaccharide (LPS)-stimulated RAW 264.7 macrophages (Liu et al., 2018). Cells were seeded in 96-well culture plates and allowed to adhere overnight. They were then divided into the following treatment groups: (1) untreated control, (2) LPS stimulated group, (3) LPS + **C17** co-treatment groups. After incubation, the cell culture supernatants were collected and levels of IL-6 and TNF-α were quantified using commercial enzyme-linked immunosorbent assay (ELISA) kits according to the manufacturers’ instructions. All experiments were performed in triplicate.

### 4.9 Membrane depolarization study

For the membrane potential assay, *S. aureus* ATCC 29213 was grown in LB broth to mid-log phase, harvested by centrifugation, washed with PBS, and adjusted to approximately 1 × 10^8^ CFU/mL in the same buffer. Aliquots (150 µL) of the cell suspension were transferred to a black 96-well plate, followed by the addition of the membrane potential-sensitive dye DiSC3(5) (10 μM, 40 µL). After 30 min of incubation in the dark at 37 °C, baseline fluorescence (excitation/emission: 622/670 nm) was recorded every 5 min over 40 min. Subsequently, 10 µL of **C17** was introduced into each well, and fluorescence monitoring continued for an additional 40 min (Koh et al., 2016). Membrane integrity was assessed using the SYTOX Green uptake assay. A bacterial suspension (150 μL, 10^8^ CFU/mL) was mixed with SYTOX Green dye (40 μL, 3 µM) in a black 96-well plate and incubated for 30 min at 37 °C in the dark. Fluorescence signals (excitation/emission: 500/530 nm) were acquired at 5-min intervals for 40 min to establish a baseline. Then, 10 µL of **C17** was added to achieve final concentrations of 64 or 256 μg/mL, and fluorescence was monitored for another 40 min.

### 4.10 Protein leakage

To evaluate the effect of compound **C17** on membrane integrity, *S. aureus* ATCC 29213 suspensions were adjusted to approximately 2 × 10^6^ CFU/mL in fresh Mueller-Hinton broth (Yang et al., 2024). The bacterial culture was treated with **C17** at final concentrations equivalent to 8×, 4×, and 1× the minimum inhibitory concentration (MIC). After incubation at 37 °C for 4 h under constant shaking, the cells were harvested by centrifugation (e.g., 10,000 × g, 10 min, 4 °C). The supernatant was carefully collected and filtered through a 0.22 μm membrane to remove any residual cells. The total protein content in the supernatant was quantified using a bicinchoninic acid (BCA) Protein Assay Kit, according to the manufacturer’s instructions. Absorbance was measured at 562 nm, and protein concentration was determined using a standard curve of bovine serum albumin.

### 4.11 ROS detection assay

The effect of compound **C17** on intracellular reactive oxygen species (ROS) accumulation in bacterial cells was determined using the fluorogenic probe 2′,7′-dichlorofluorescein diacetate (DCFH-DA) (Yang et al., 2024). Briefly, frozen bacterial stocks were subcultured in Mueller-Hinton broth and grown with shaking at 37 °C until mid-logarithmic phase. The cells were then harvested by centrifugation, washed thoroughly with phosphate-buffered saline (PBS), and adjusted to an optical density (OD_600_) of 0.5. Subsequently, the bacterial suspension was incubated with 10 µM DCFH-DA at 37 °C for 20 min in the dark to facilitate probe uptake and hydrolysis. After labeling, the cells were washed again to remove excess dye and resuspended in PBS. Aliquots of 190 µL of the labeled suspension were dispensed into a black 96-well plate, and 10 µL of **C17** at desired concentrations was added to each well. Following 30 min of incubation at 37 °C, fluorescence was quantified (excitation/emission = 488/525 nm) using a microplate reader.

### 4.12 Plasma protein binding rate of **C17**

The plasma protein binding of **C17** was assessed using equilibrium dialysis. Pre-hydrated dialysis bags were sealed at one end and filled with 0.5 mL of blank plasma from Sprague-Dawley rats, then securely closed. Each bag was immersed in a centrifuge tube containing 40 mL of PBS dialysate supplemented with 30 μM **C17**. The system was incubated at 37 °C with continuous shaking at 100 rpm for 24 h to reach equilibrium. Subsequently, samples from both the plasma and dialysate compartments were collected and analyzed via HPLC. The plasma protein binding rate was calculated using the formula: Binding (%) = [(A − B)/A] × 100%, where A represents the total drug concentration inside the bag and B denotes the free drug concentration in the dialysate (Di, 2021; Xiao et al., 2023). All experiments included nonspecific binding controls and were conducted in triplicate.

### 4.13 Determination of logD_7.4_ for **C17**

The logD_7_._4_ value of compound **C17** was determined using the shake-flask method (Bruneau and McElroy, 2006; Wang Y. et al., 2023). Briefly, 1 mg of **C17** was dissolved in 2 mL of pH 7.4 buffer-saturated n-octanol, mixed with an equal volume of n-octanol-saturated buffer, and vortexed for 3 min. The mixture was then equilibrated at 37 °C with shaking at 200 rpm for 24 h. After centrifugation and phase separation, aliquots from both the octanol and aqueous phases were diluted in methanol (dilution factors: x for octanol, y for buffer) and analyzed by HPLC. LogD_7.4_ was calculated as log [(peak area in octanol × x)/(peak area in buffer × y)]. All measurements were performed in triplicate.

### 4.14 Liver microsomal stability assay for **C17**

The metabolic stability of compound **C17** was evaluated in rat liver microsomes (Li et al., 2022). Incubation mixtures contained 0.5 mL of liver microsomes (20 mg protein/mL), 100 μM of compound **C17**, and 20 mM NADPH in 0.1 M phosphate-buffered saline (PBS, pH 7.4). The reaction was conducted at 37 °C and aliquots were quenched with ice-cold acetonitrile at predetermined time points over a 120-min period. Following quenching, samples were vortexed for 30 s and centrifuged at 12,000 rpm for 15 min at 4 °C. The resulting supernatants were evaporated under a gentle stream of nitrogen gas, reconstituted in 200 μL of methanol, and subjected to HPLC analysis. All samples were processed in triplicate to ensure reproducibility. The percentage of the parent compound remaining at each time point was quantified to assess metabolic stability.

### 4.15 Statistical analysis

All values are expressed as the mean ± standard error of the mean (SEM) from a minimum of three independent experiments. Statistical analyses were performed using SPSS 21.0 software. Differences between groups were assessed by one-way analysis of variance (ANOVA).

## Data Availability

The original contributions presented in the study are included in the article/Supplementary Material, further inquiries can be directed to the corresponding author.
