# Supplementary material for: Design, synthesis, and biological evaluation of derivatives from p-toluic hydrazide: lead compound C17 exhibits membrane-disrupting and anti-biofilm activities against Staphylococcus aureus and other gram-positive bacteria
Source: Front Chem. 2026 May 14;14:1825790. doi: 10.3389/fchem.2026.1825790 (PMC13216026; doi:10.3389/fchem.2026.1825790)
Supplement: Supplementary file 1 [file DataSheet1.doc]

**Design, Synthesis, and Biological Evaluation of Derivatives from p‑Toluic Hydrazide: Lead Compound C17 Exhibits Membrane‑Disrupting and Anti‑biofilm Activities against *Staphylococcus aureus* and Other Gram‑Positive Bacteria**

Yaguang Liu^1*^, Lianzhi Hu[[1]](#footnote-2), Binbin Liu^1^, Zheng Qu^1^

*^1^The Second Hospital of QinHuangDao, Qinhuangdao, China, 066000*

**Table of Contents**

*^1^H and ^13^C NMR of* **C1**····················································································1

*^1^H and ^13^C NMR of* **C2**····················································································2

*^1^H and ^13^C NMR of* **C3**····················································································3

*^1^H and ^13^C NMR of* **C4**····················································································4

*^1^H and ^13^C NMR of* **C5**····················································································5

*^1^H and ^13^C NMR of* **C6**····················································································6

*^1^H and ^13^C NMR of* **C7**····················································································7

*^1^H and ^13^C NMR of* **C8**····················································································8

*^1^H and ^13^C NMR of* **C9**····················································································9

*^1^H and ^13^C NMR of* **C10**··················································································10

*^1^H and ^13^C NMR of* **C11**··················································································11

*^1^H and ^13^C NMR of* **C12**··················································································12

*^1^H and ^13^C NMR of* **C13**··················································································13

*^1^H and ^13^C NMR of* **C14**··················································································14

*^1^H and ^13^C NMR of* **C15**··················································································15

*^1^H and ^13^C NMR of* **C16**··················································································16

*^1^H and ^13^C NMR of* **C17**··················································································17

*Mass spectrum and HPLC purity chromatogram of* **C17**····································18

*^1^H and ^13^C NMR of* **C18**··················································································19


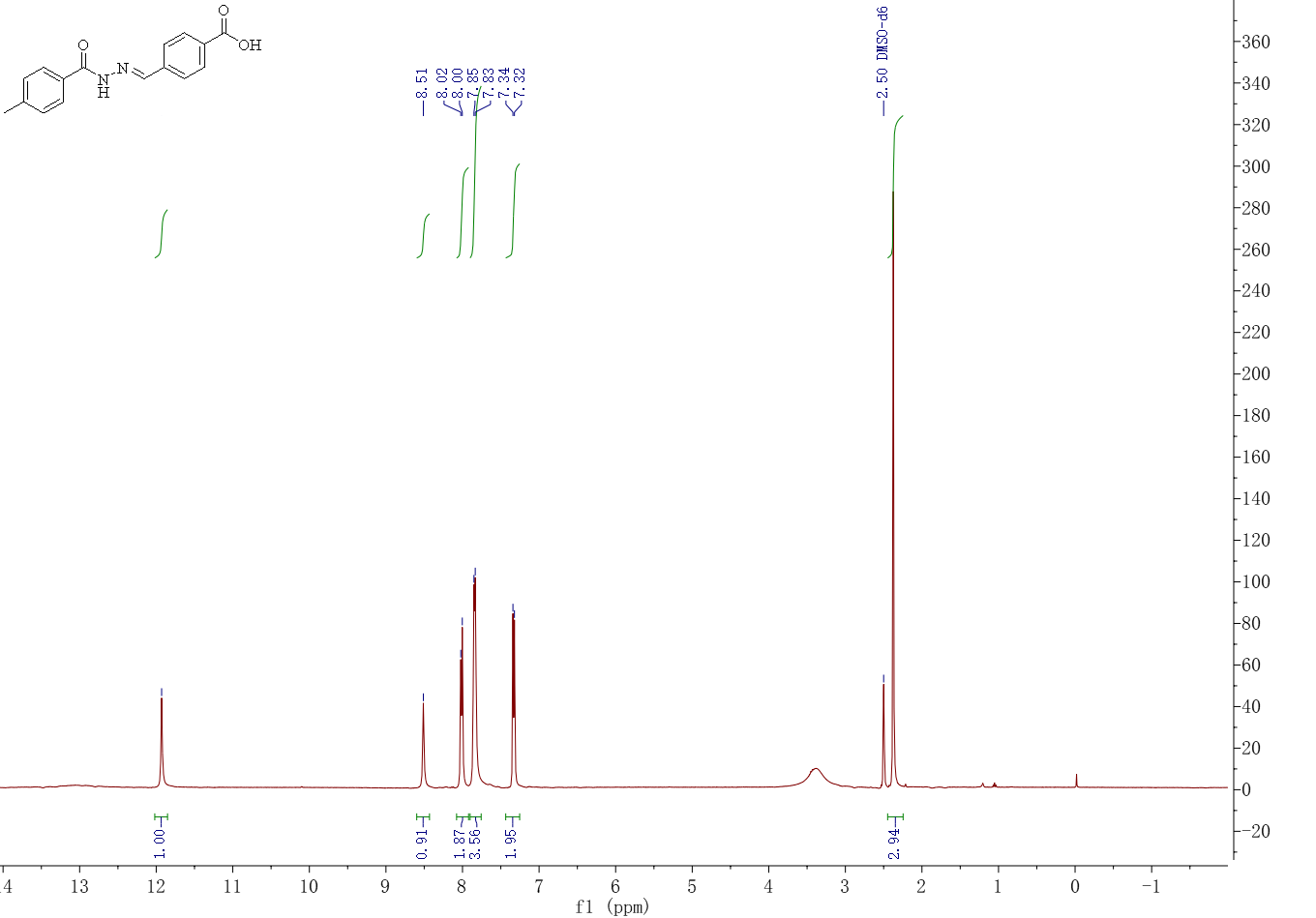


Fig 1. *^1^H NMR of* **C1** (400 MHz, DMSO)


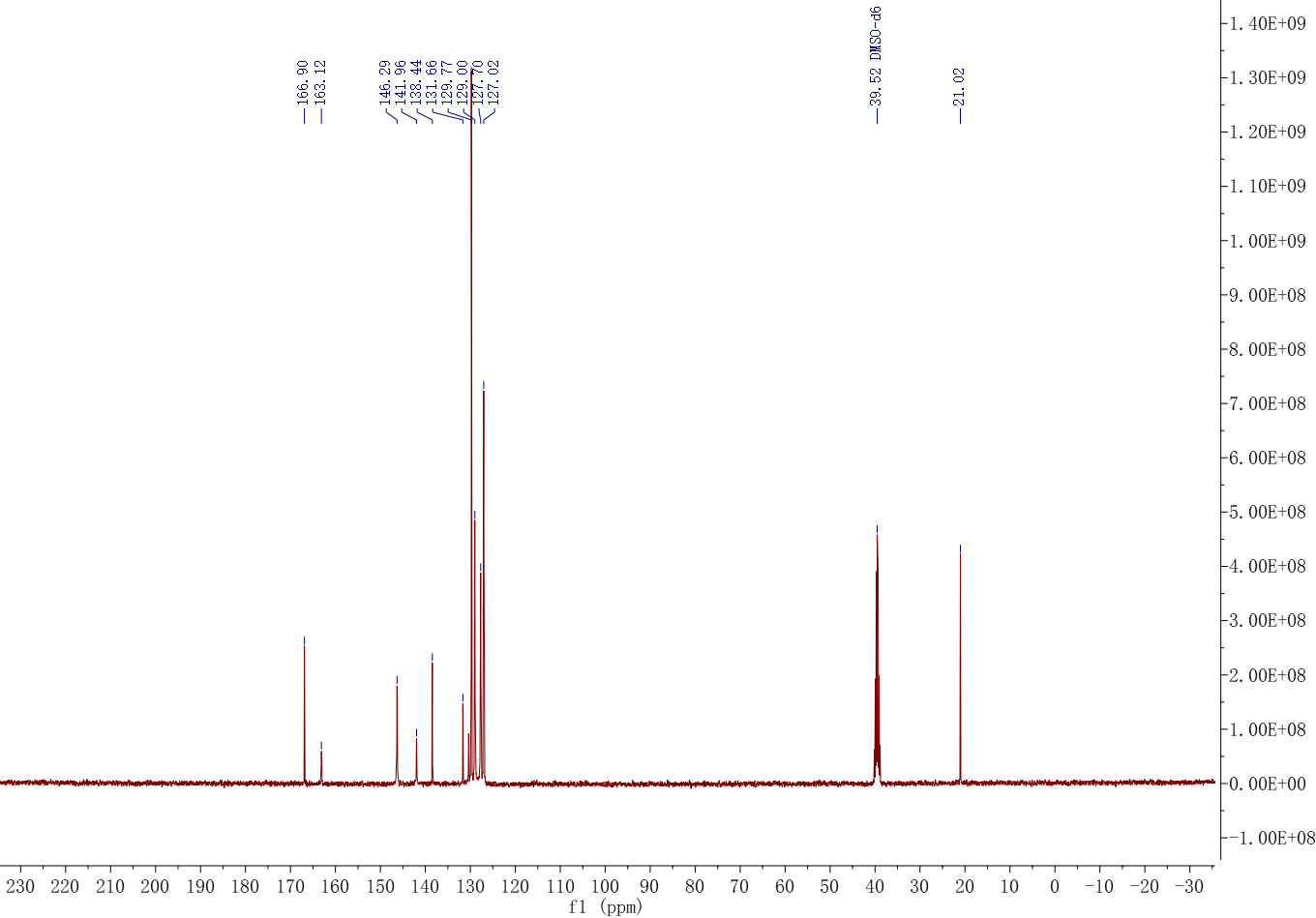


Fig 2. *^13^C NMR of* **C1** (100 MHz, DMSO)


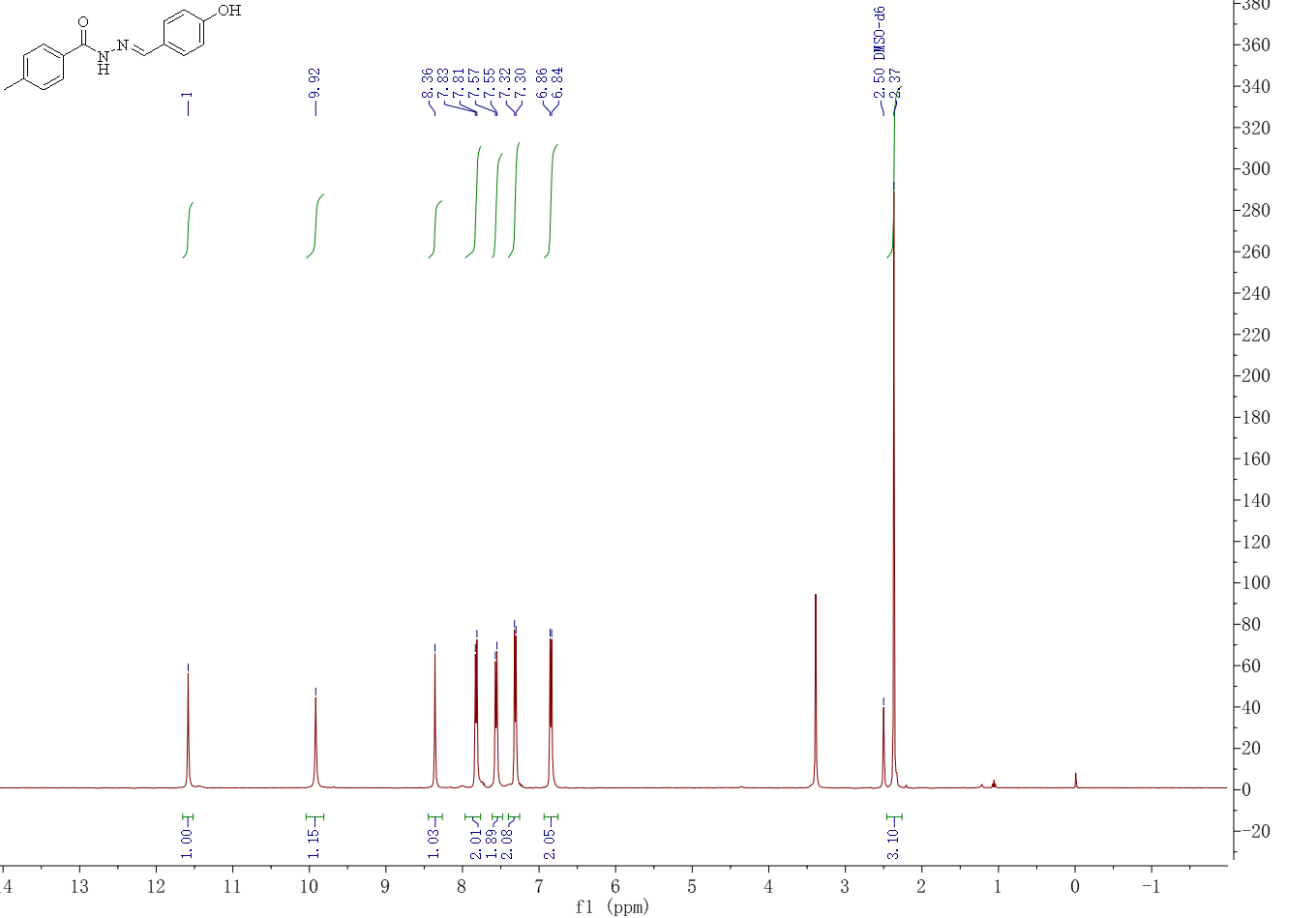


Fig 3. *^1^H NMR of* **C2** (400 MHz, DMSO)


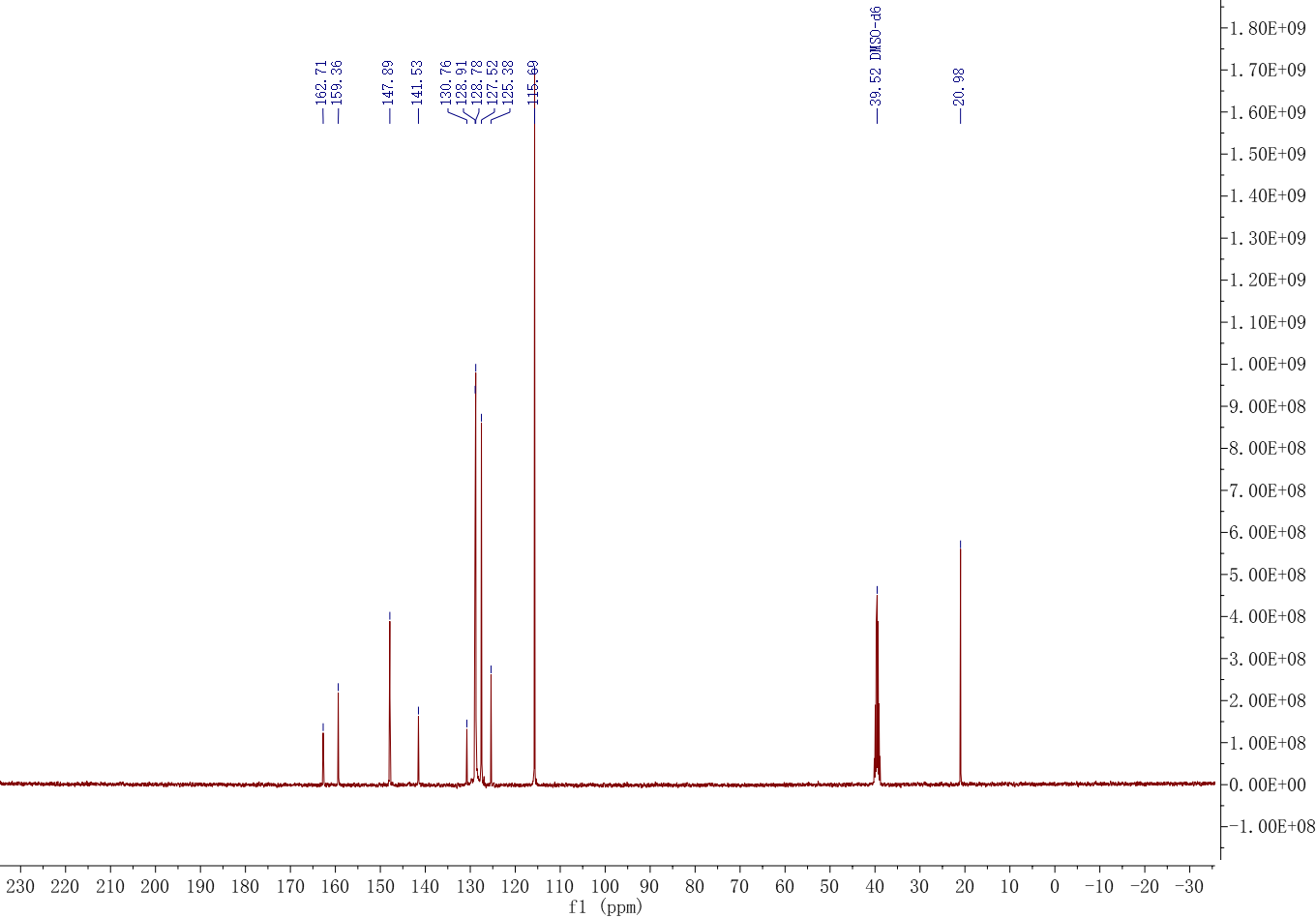


Fig 4. *^13^C NMR of* **C2** (100 MHz, DMSO)


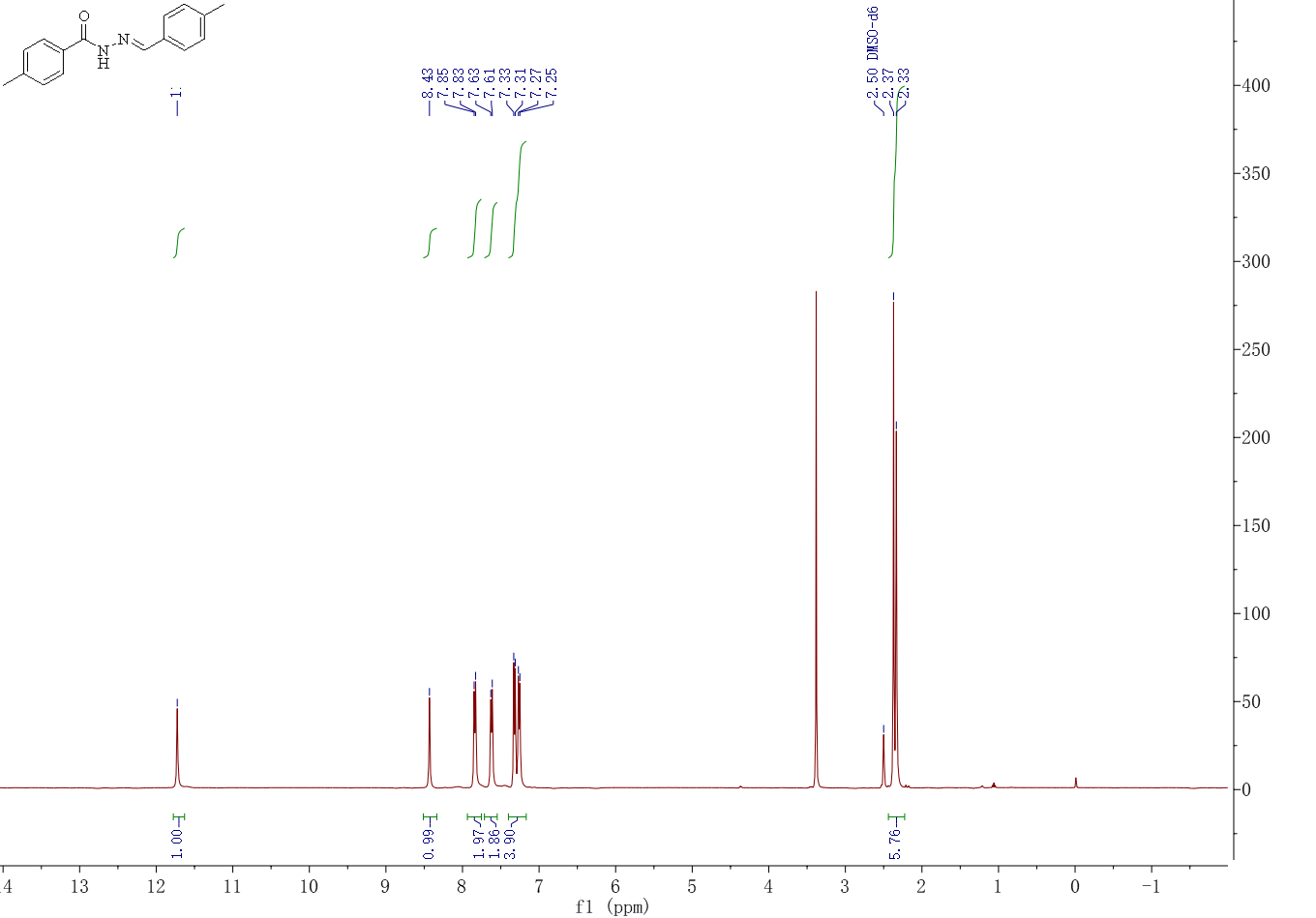


Fig 5. *^1^H NMR of* **C3** (400 MHz, DMSO)


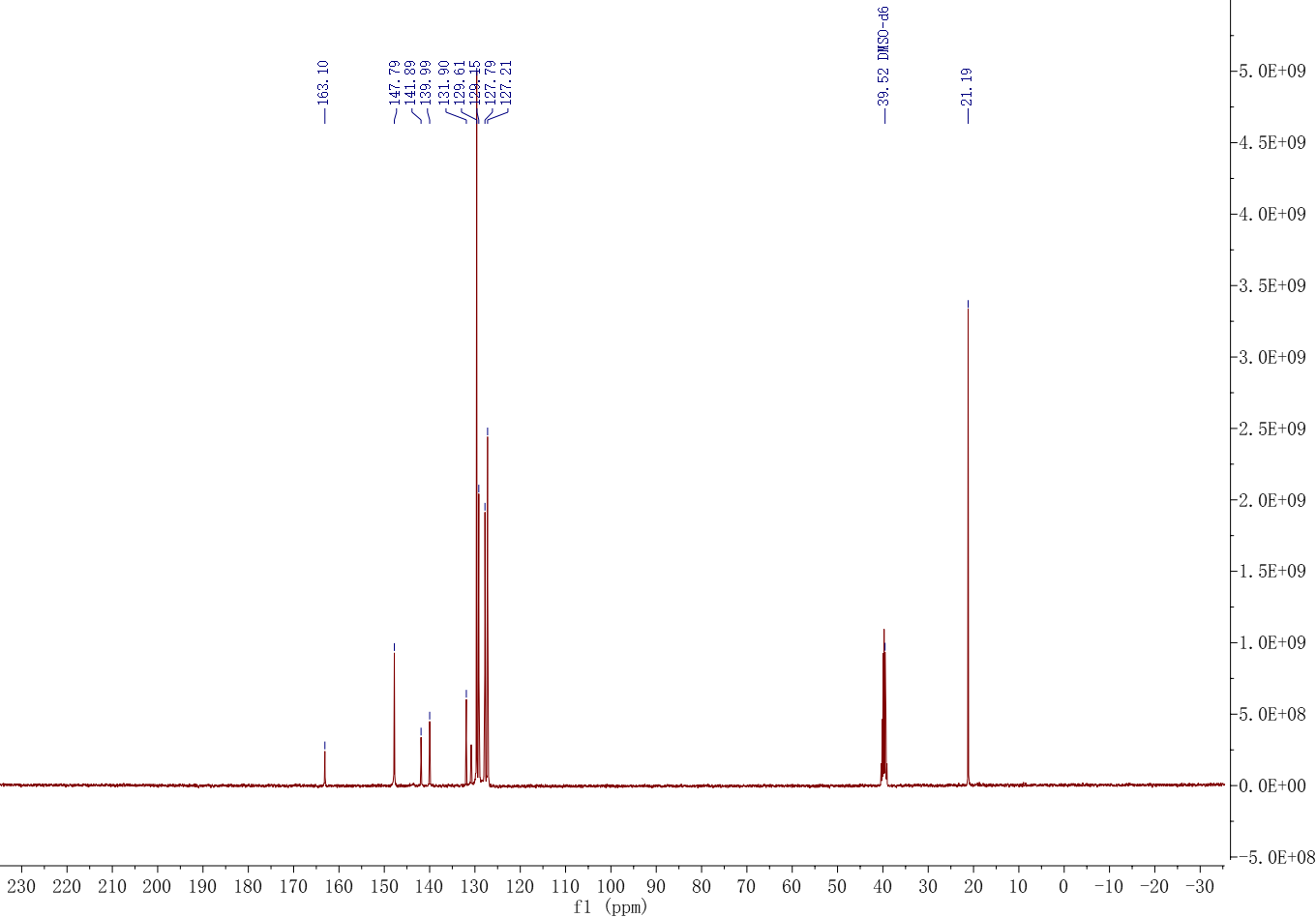


Fig 6. *^13^C NMR of* **C3** (100 MHz, DMSO)


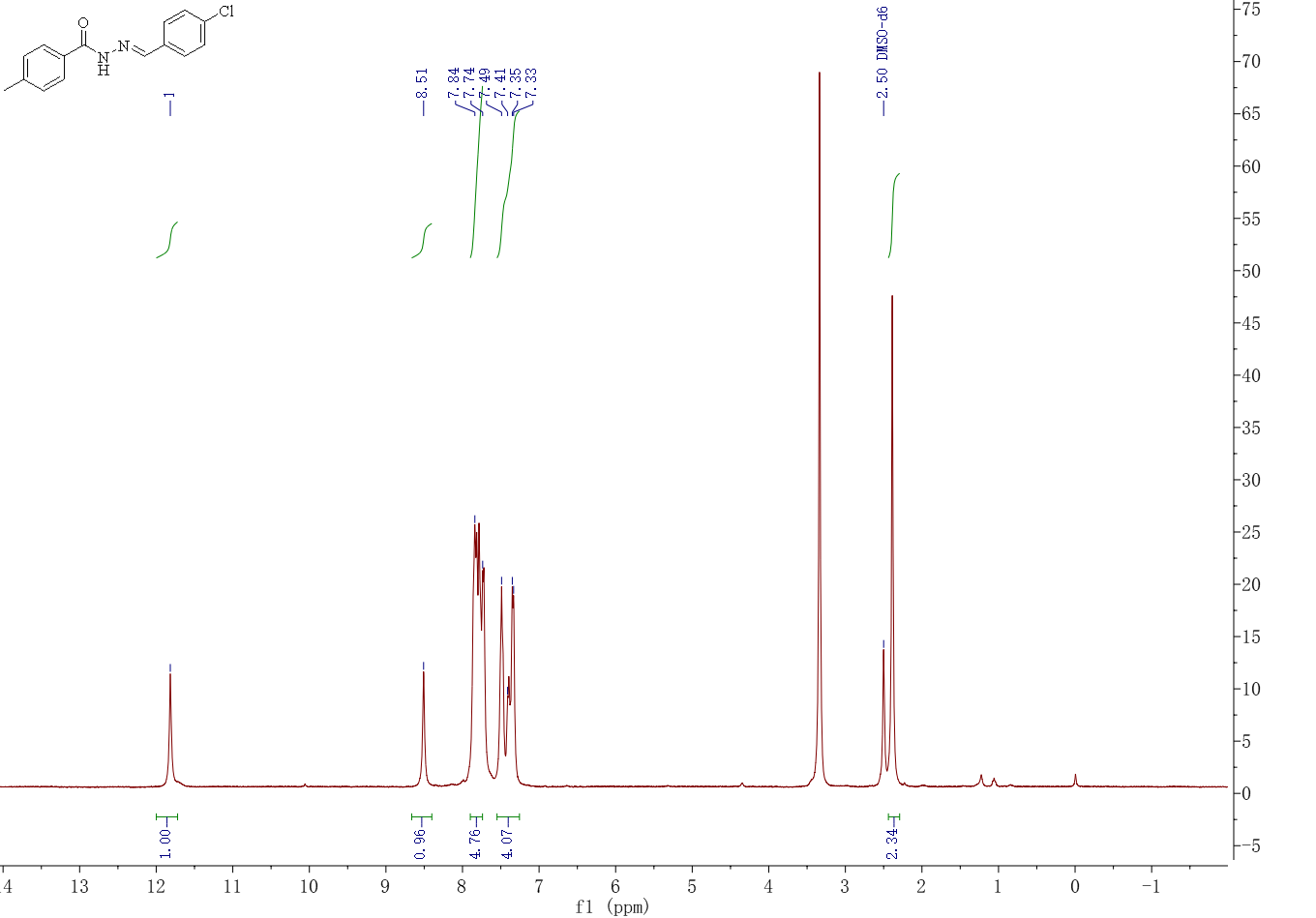


Fig 7. *^1^H NMR of* **C4** (400 MHz, DMSO)


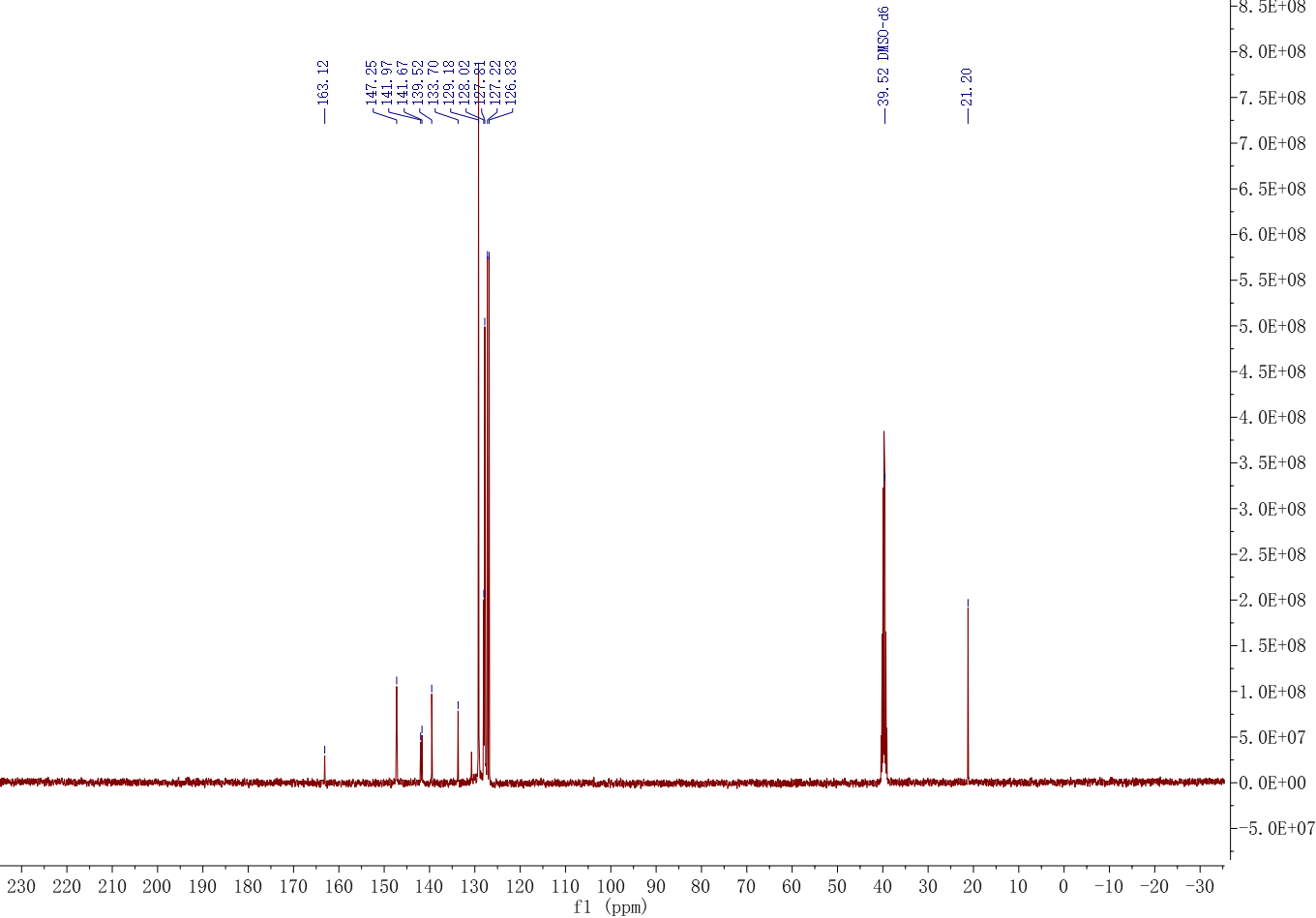


Fig 8. *^13^C NMR of* **C4** (100 MHz, DMSO)


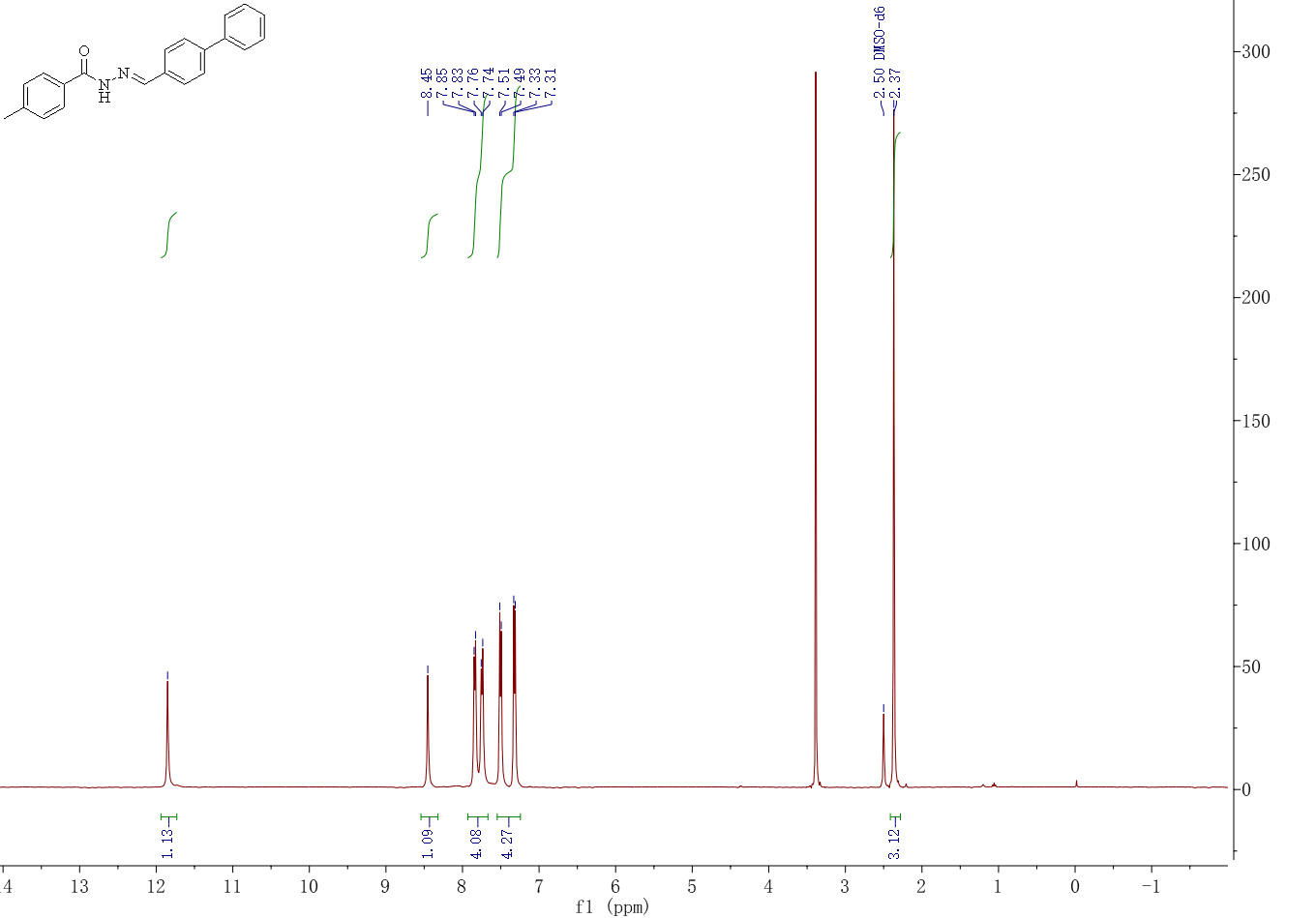


Fig 9. *^1^H NMR of* **C5** (400 MHz, DMSO)


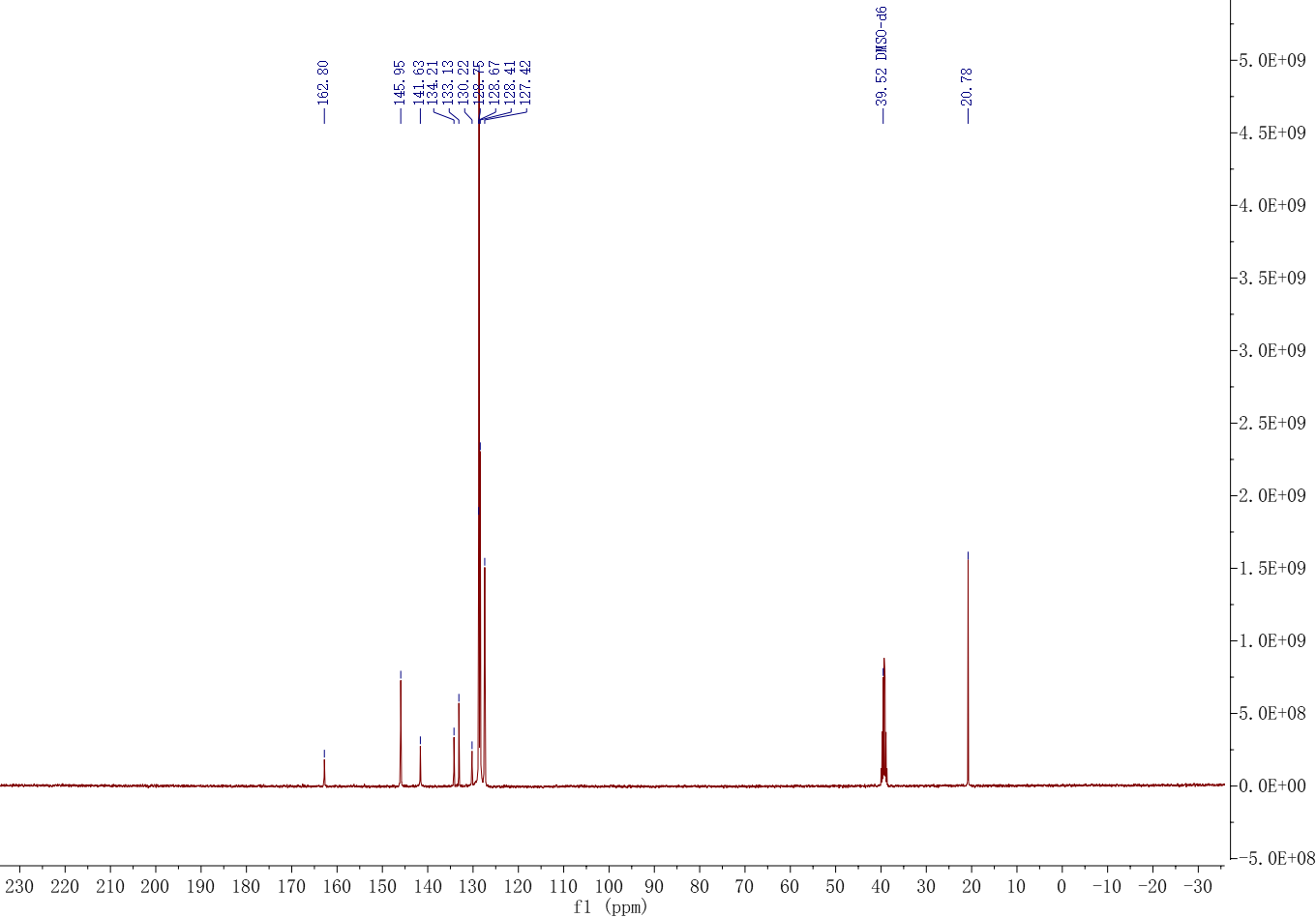


Fig 10. *^13^C NMR of* **C5** (100 MHz, DMSO)


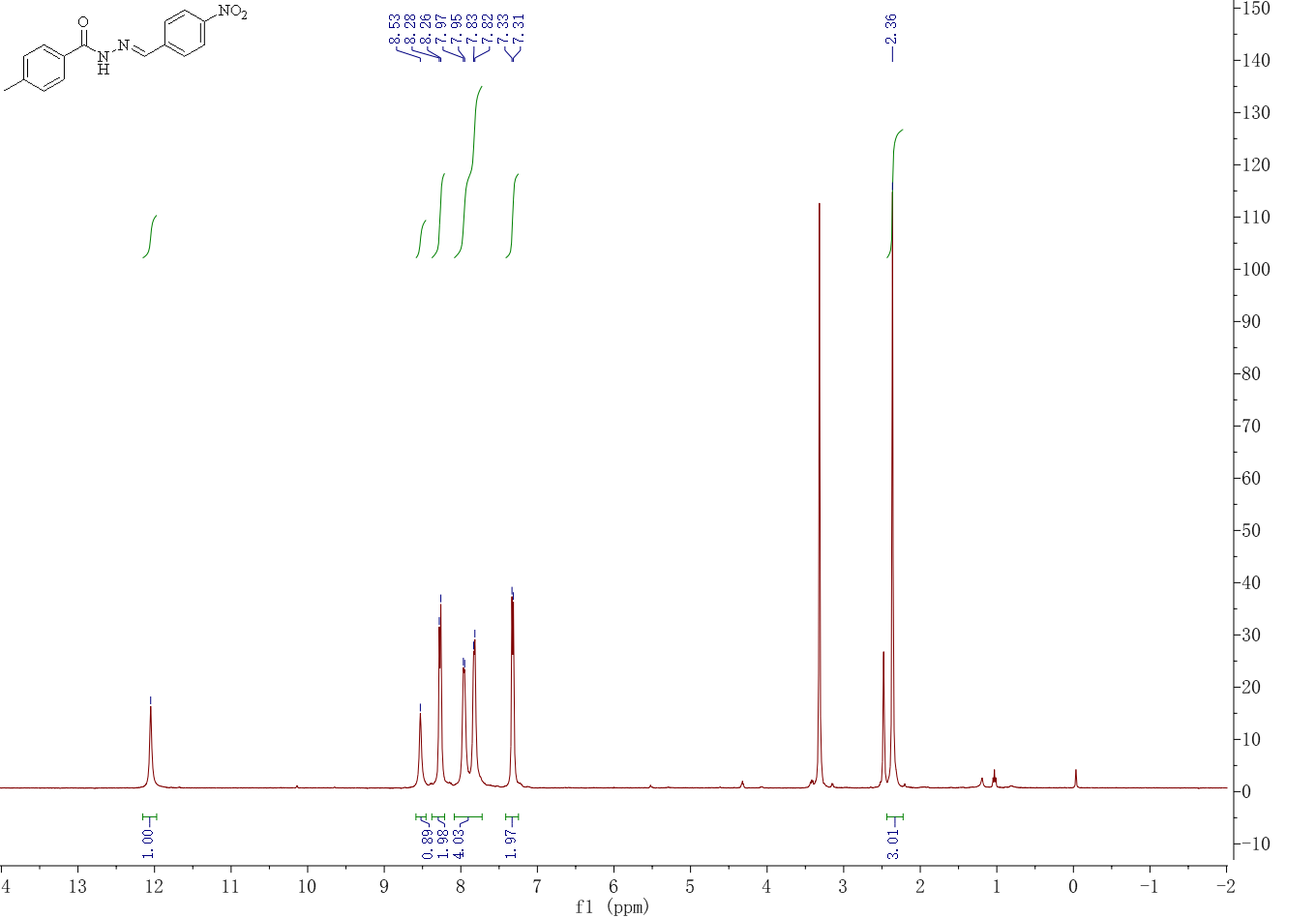


Fig 11. *^1^H NMR of* **C6** (400 MHz, DMSO)


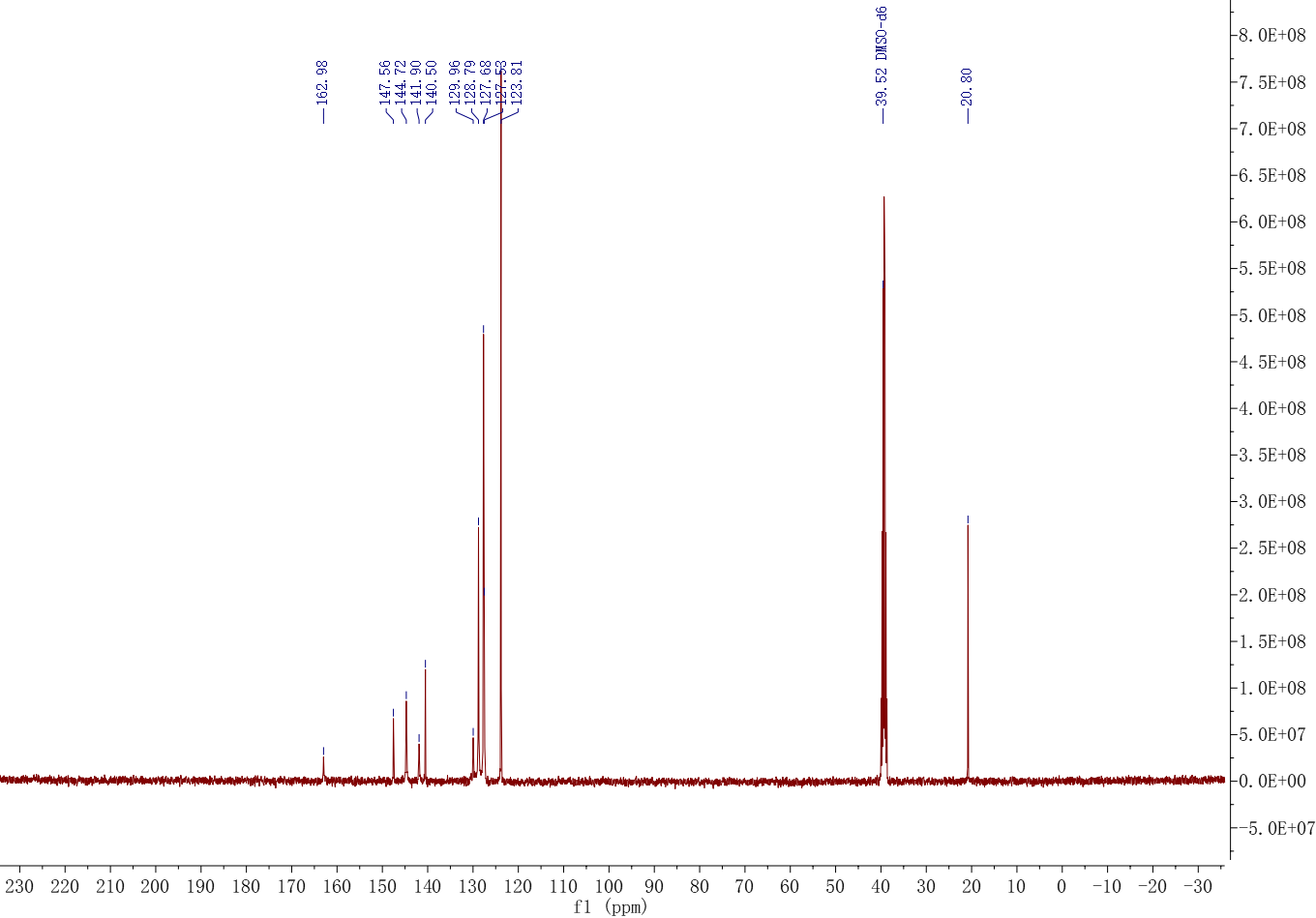


Fig 12. *^13^C NMR of* **C6** (100 MHz, DMSO)


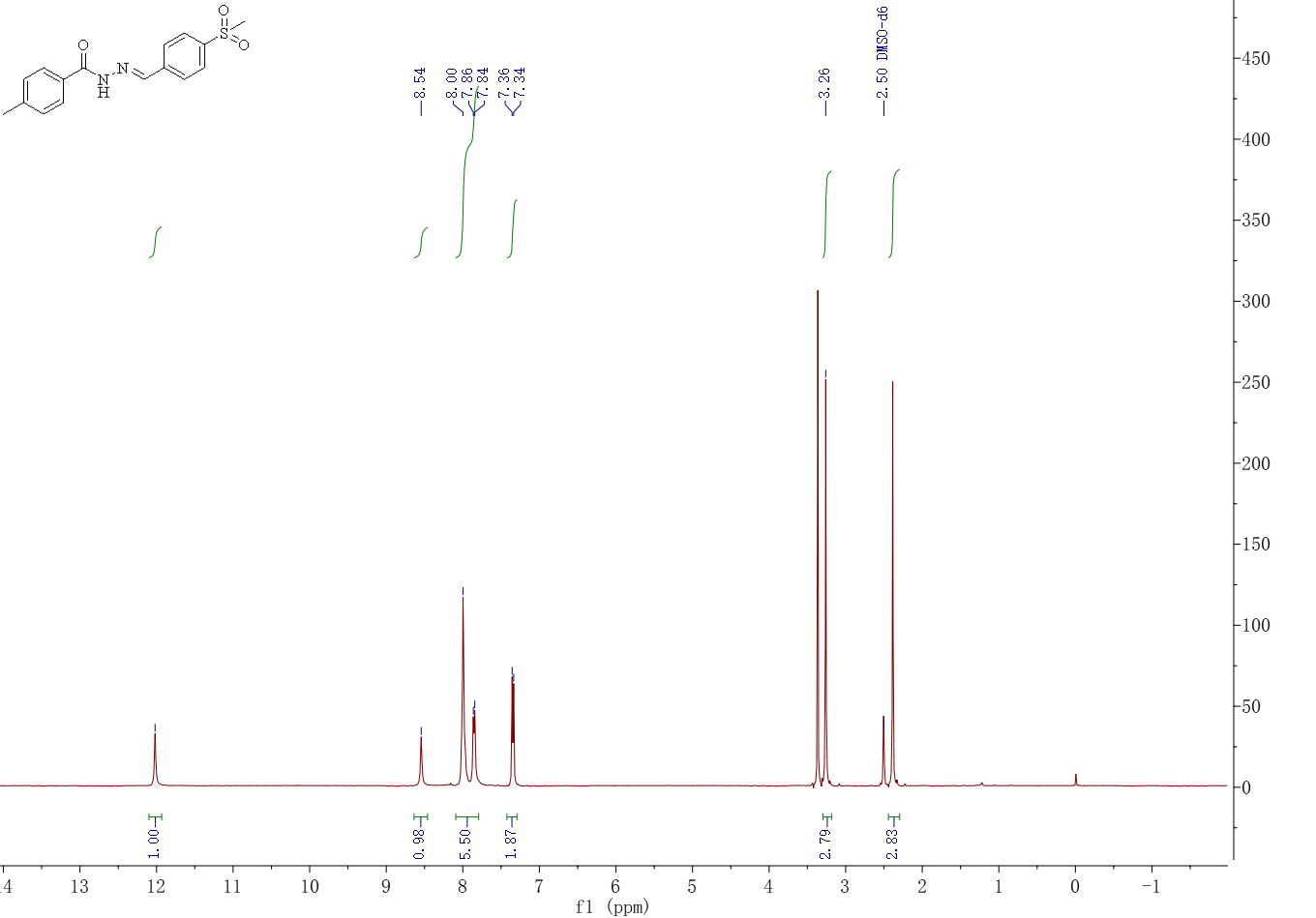


Fig 13. *^1^H NMR of* **C7** (400 MHz, DMSO)


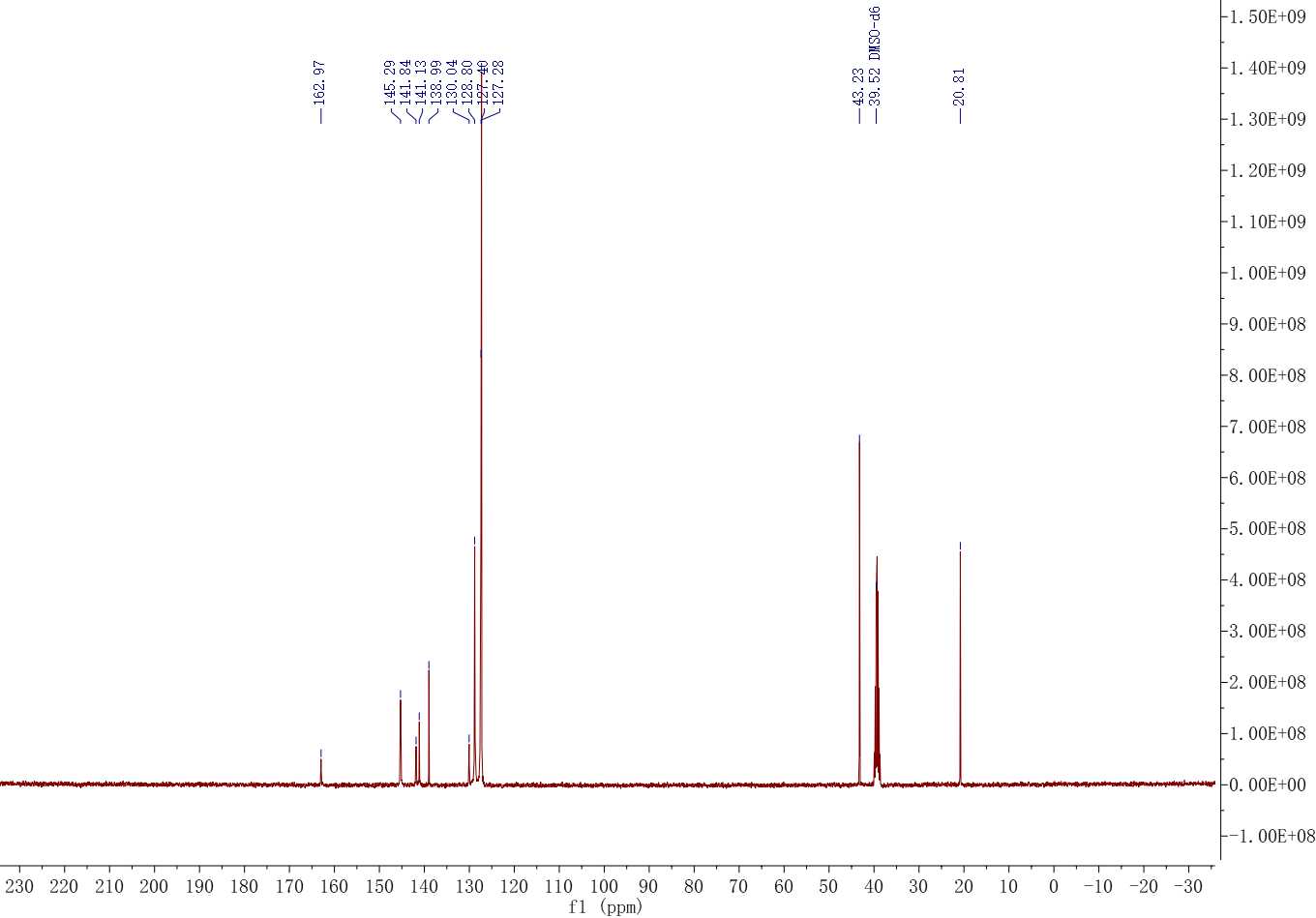


Fig 14. *^13^C NMR of* **C7** (100 MHz, DMSO)


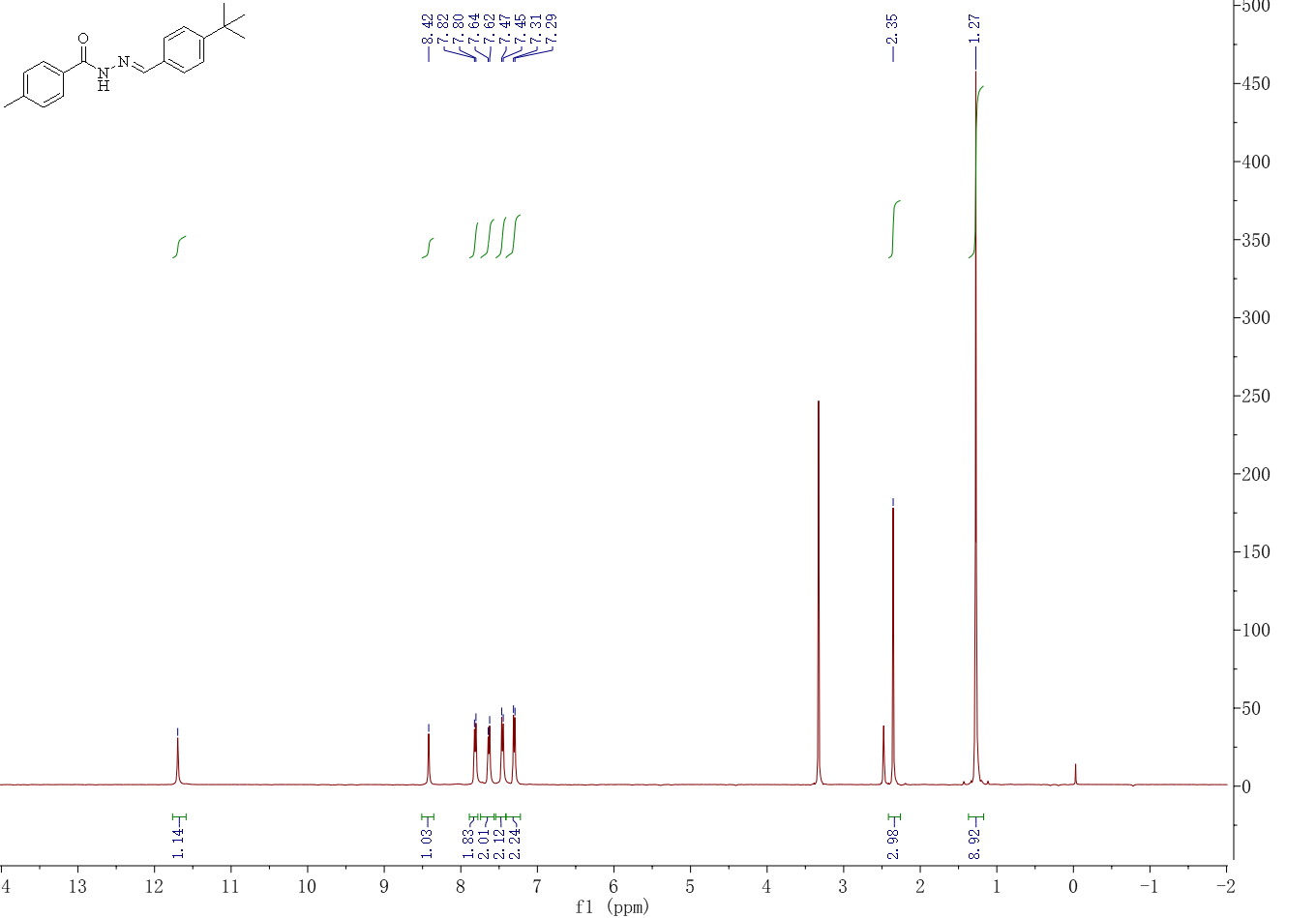


Fig 15. *^1^H NMR of* **C8** (400 MHz, DMSO)


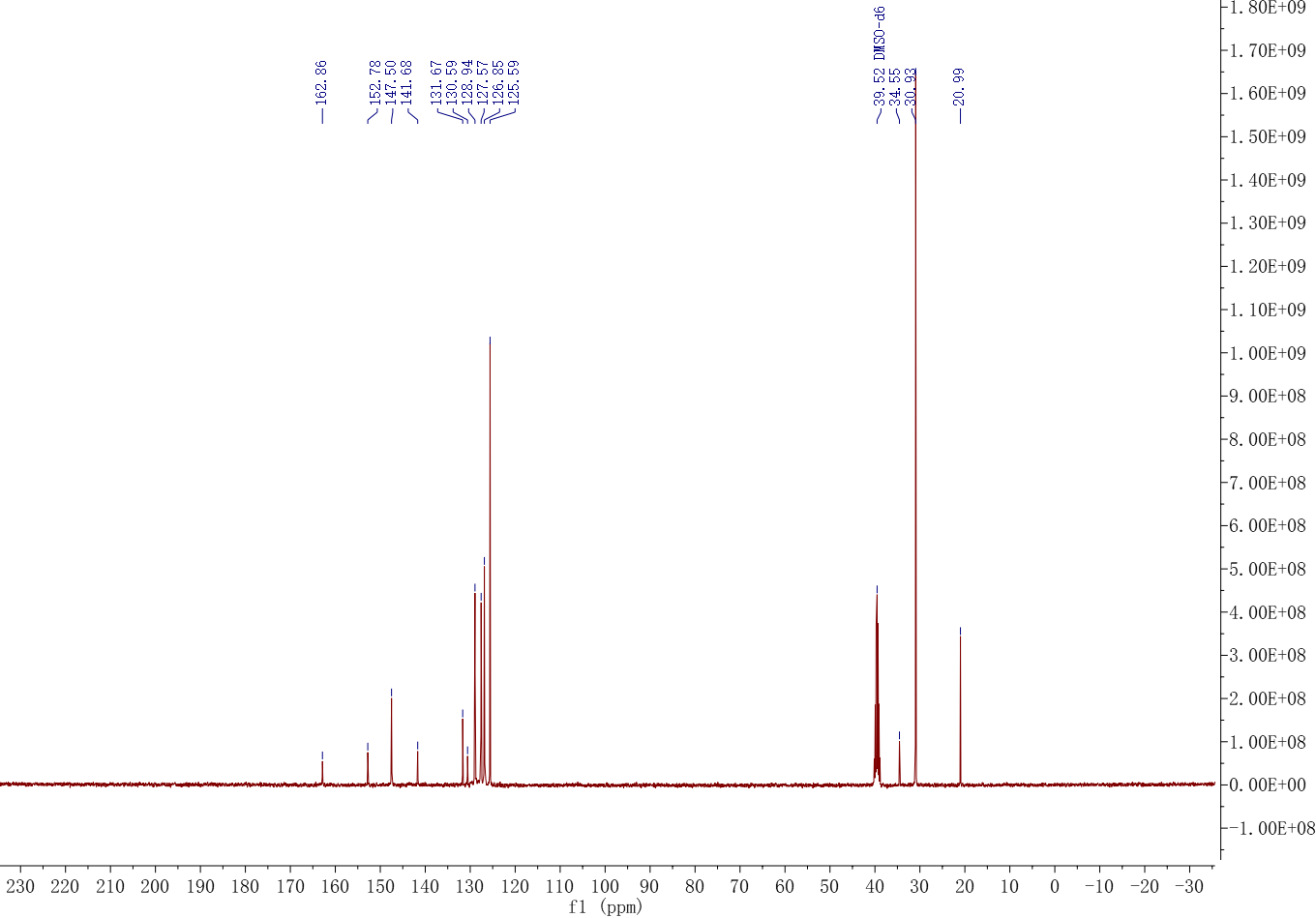


Fig 16. *^13^C NMR of* **C8** (100 MHz, DMSO)


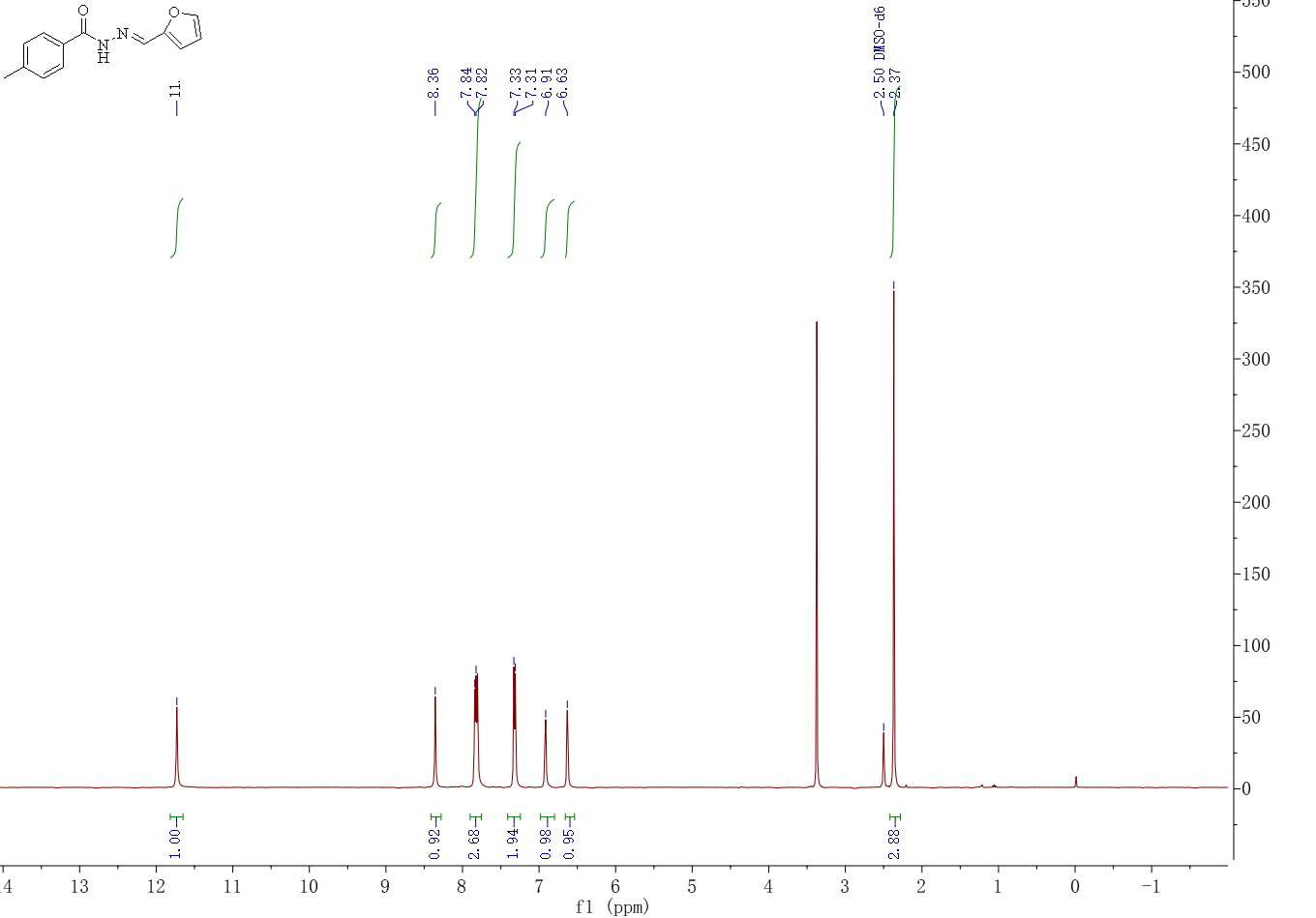


Fig 17. *^1^H NMR of* **C9** (400 MHz, DMSO)


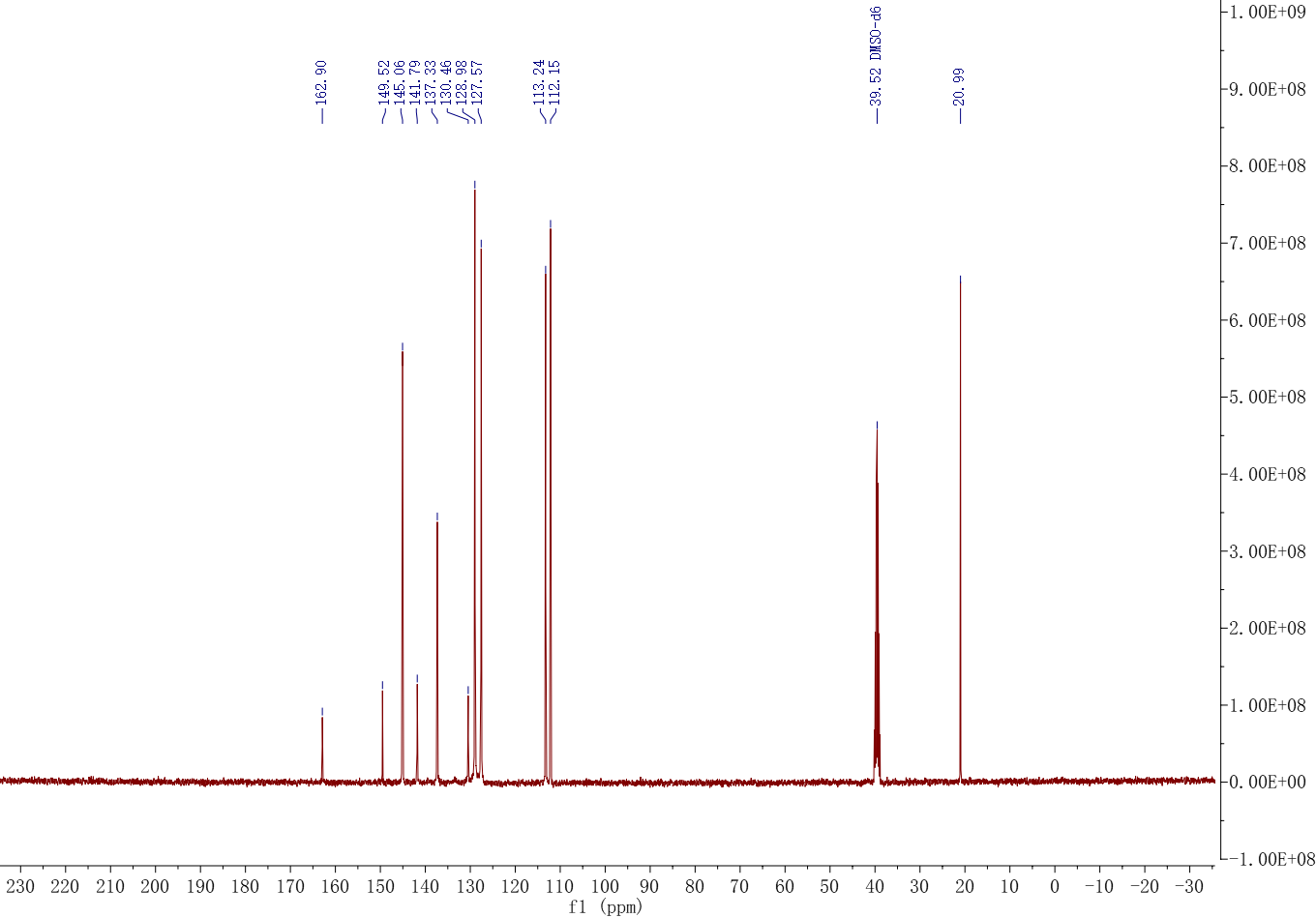


Fig 18. *^13^C NMR of* **C9** (100 MHz, DMSO)


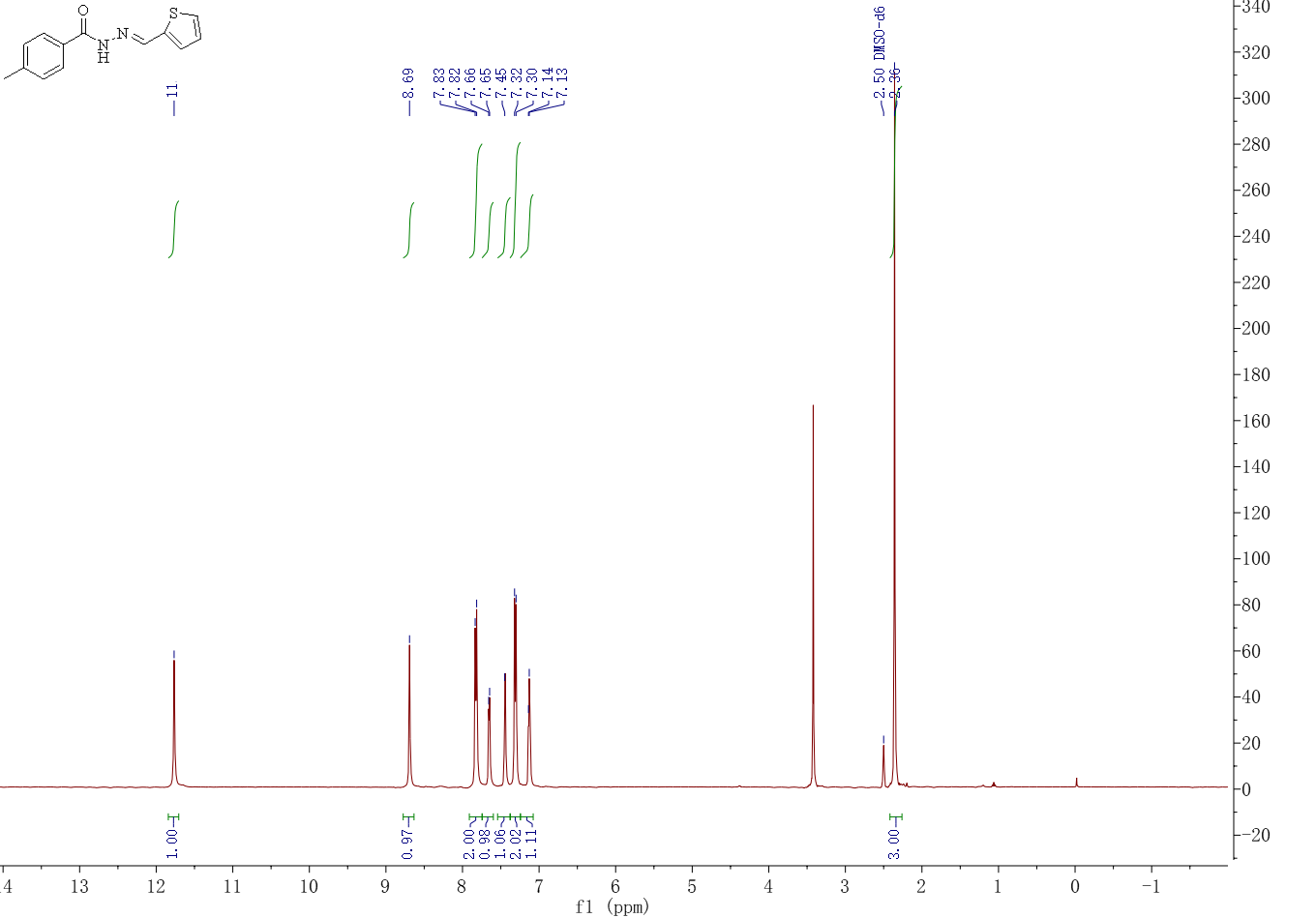


Fig 19. *^1^H NMR of* **C10** (400 MHz, DMSO)


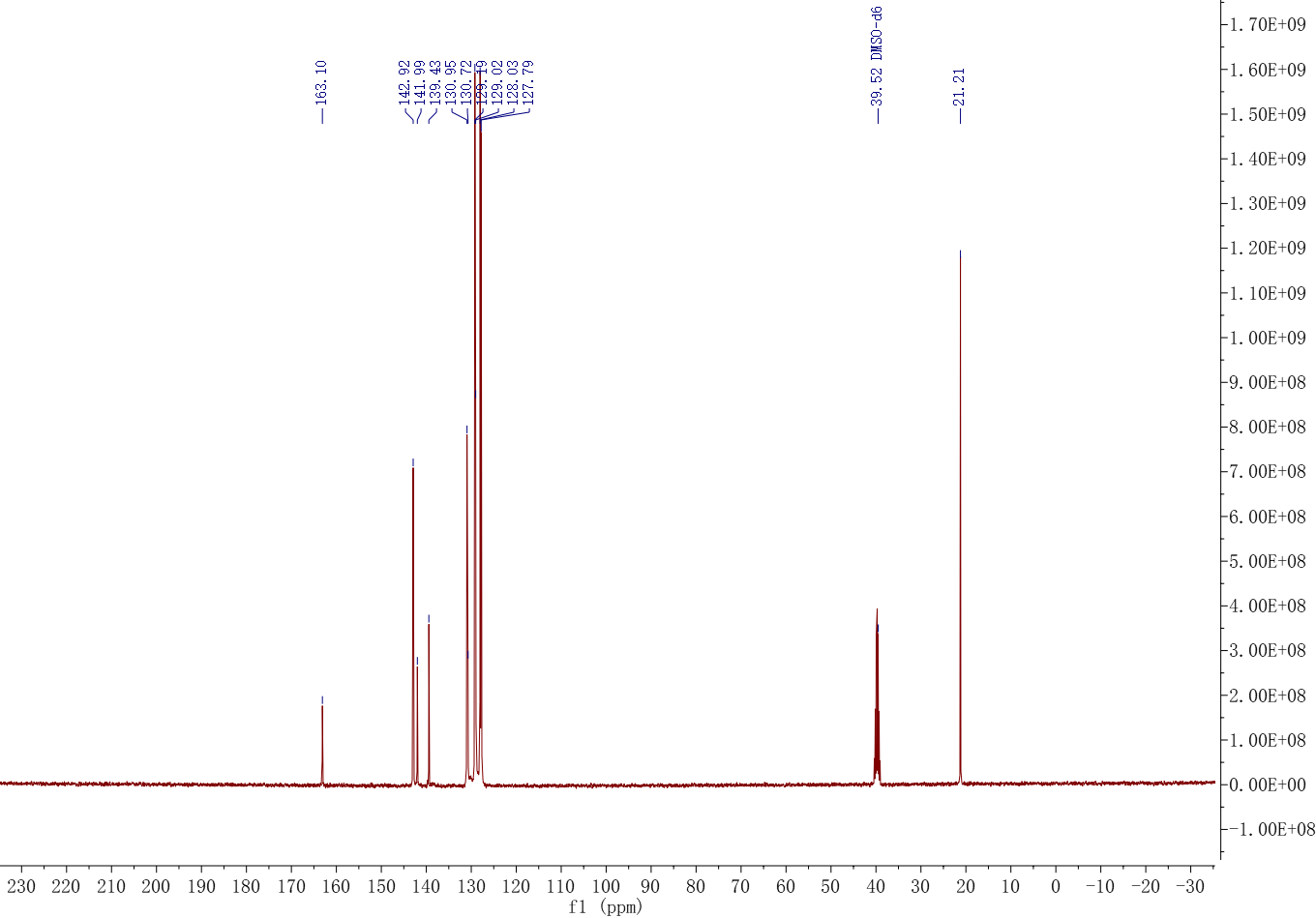


Fig 20. *^13^C NMR of* **C10** (100 MHz, DMSO)


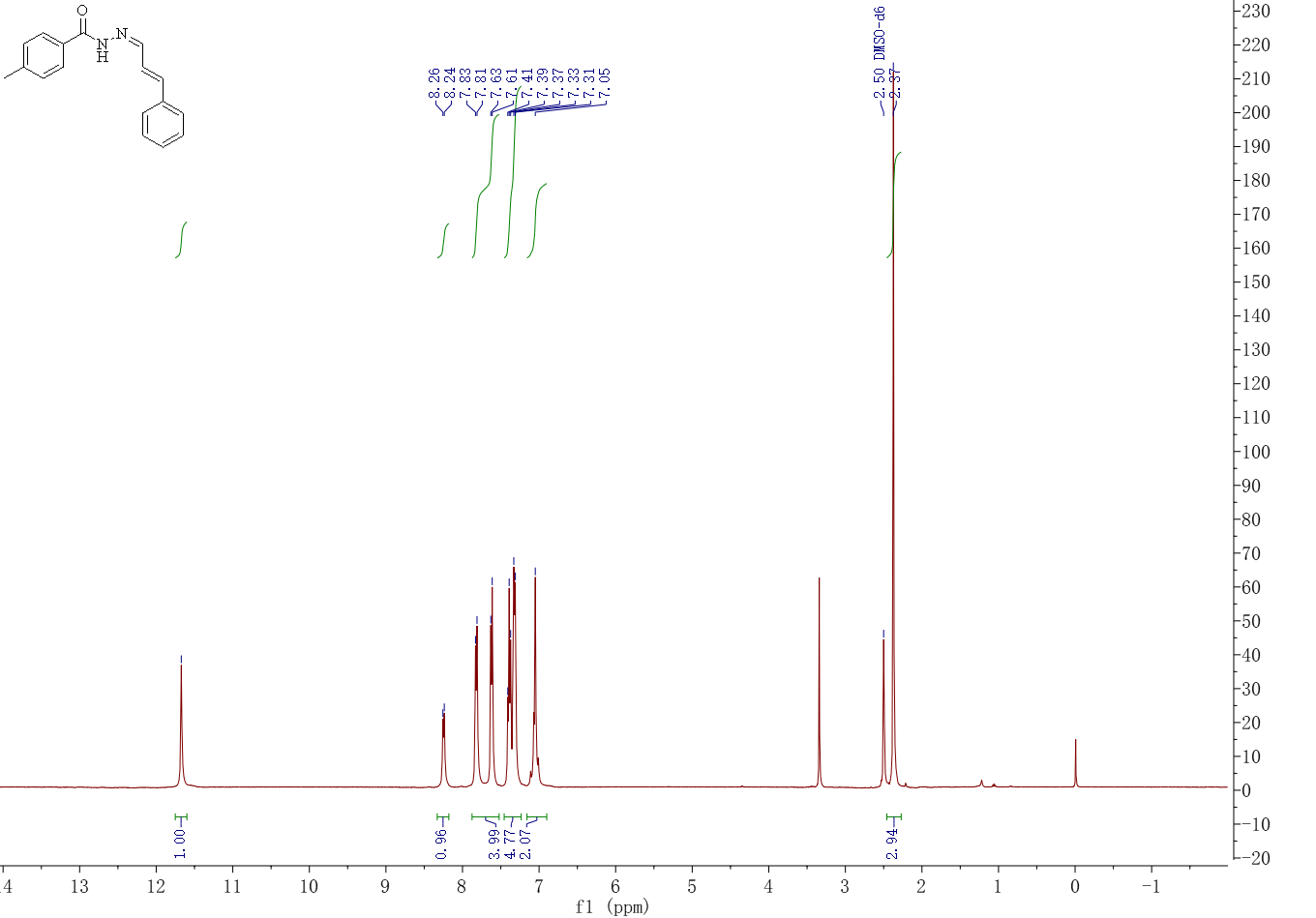


Fig 21. *^1^H NMR of* **C11** (400 MHz, DMSO)


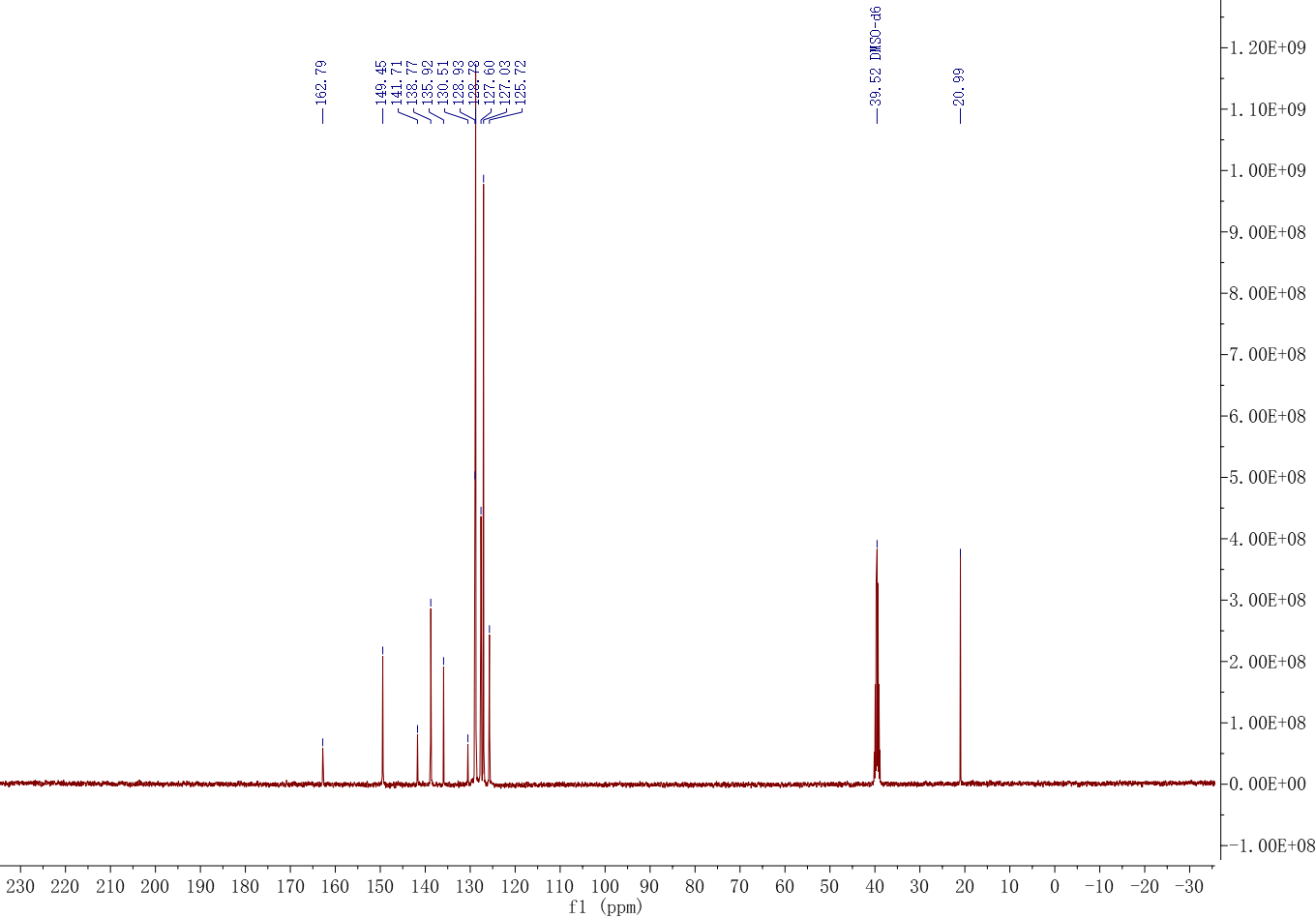


Fig 22. *^13^C NMR of* **C11** (100 MHz, DMSO)


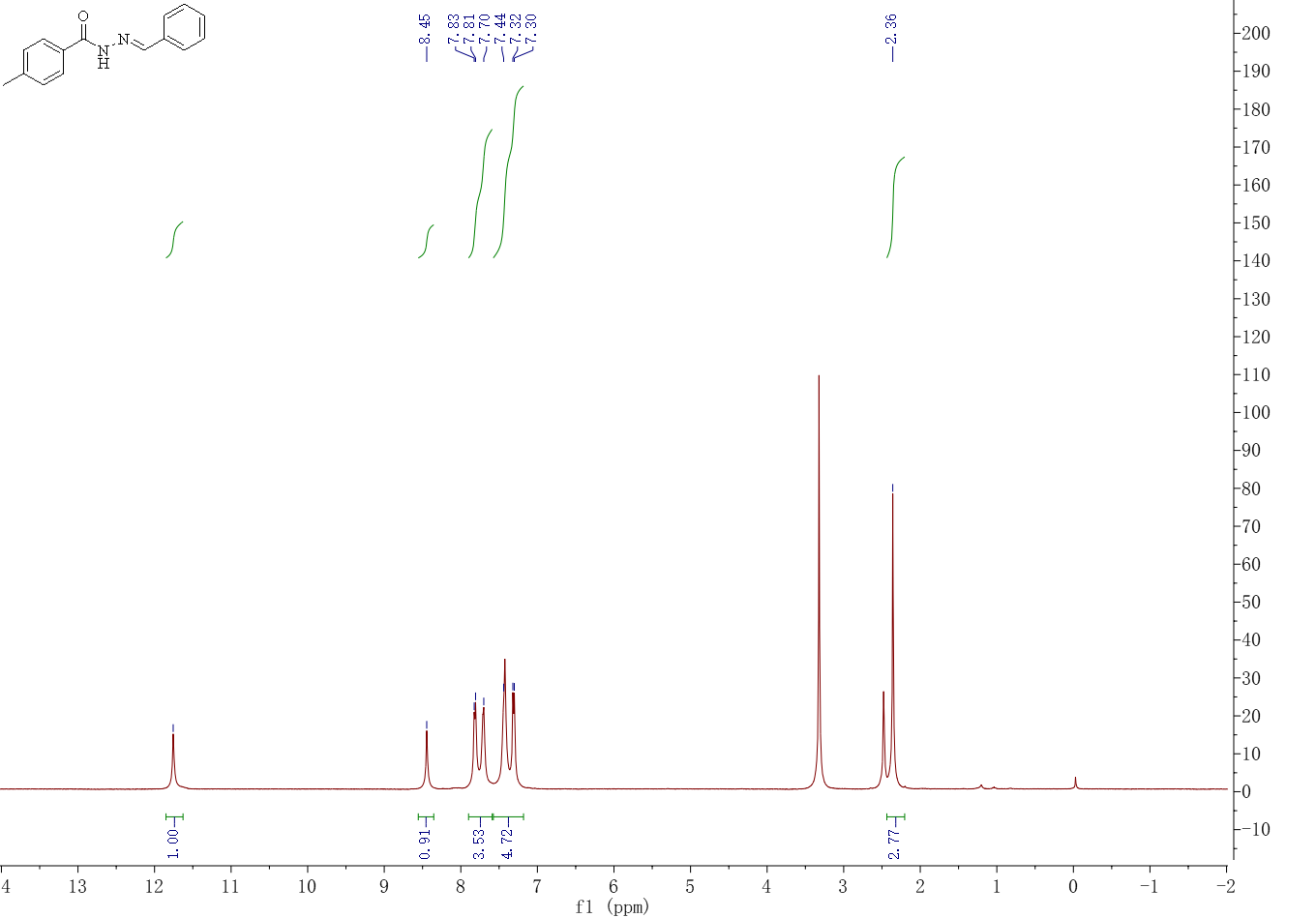


Fig 23. *^1^H NMR of* **C12** (400 MHz, DMSO)


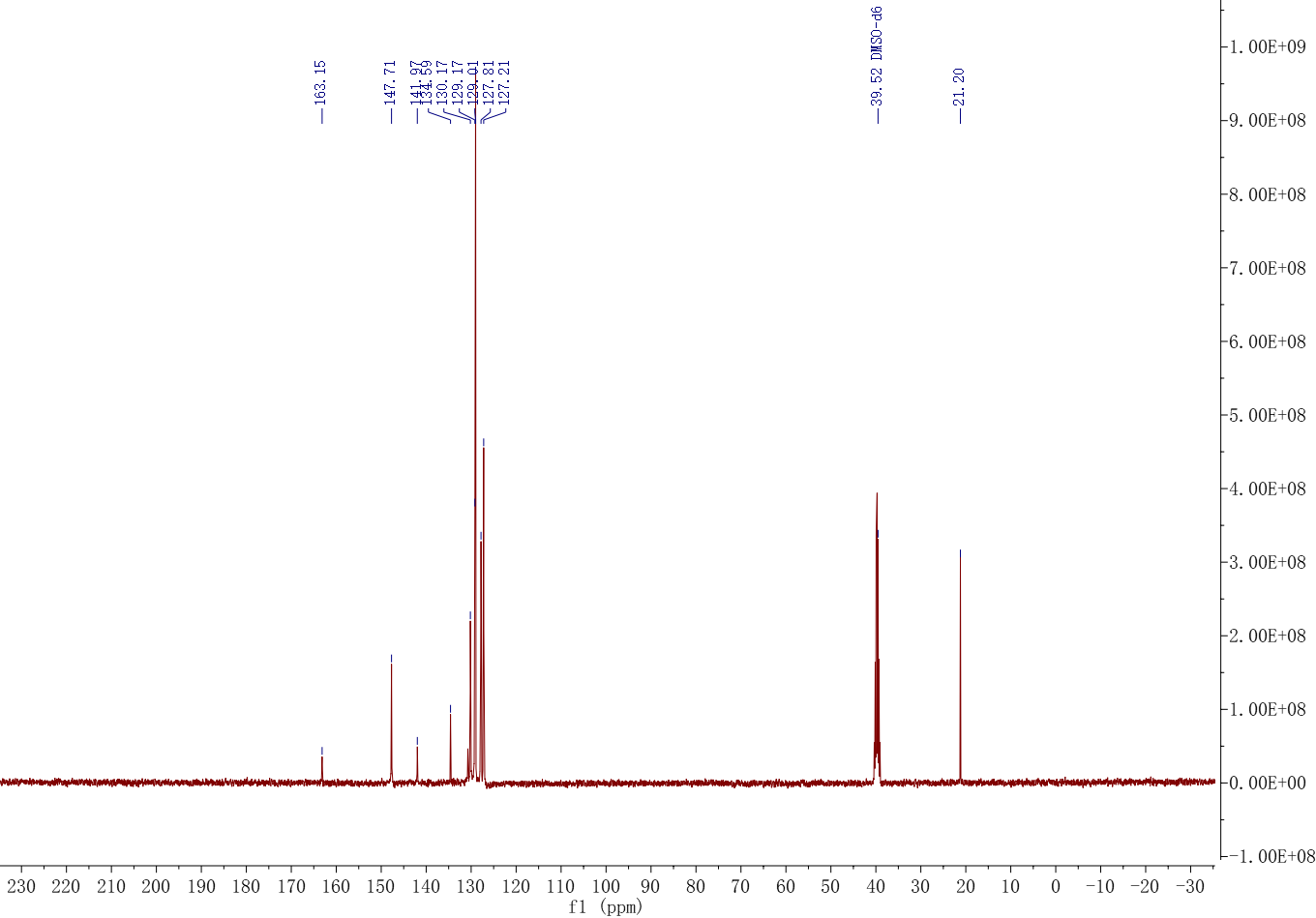


Fig 24. *^13^C NMR of* **C12** (100 MHz, DMSO)


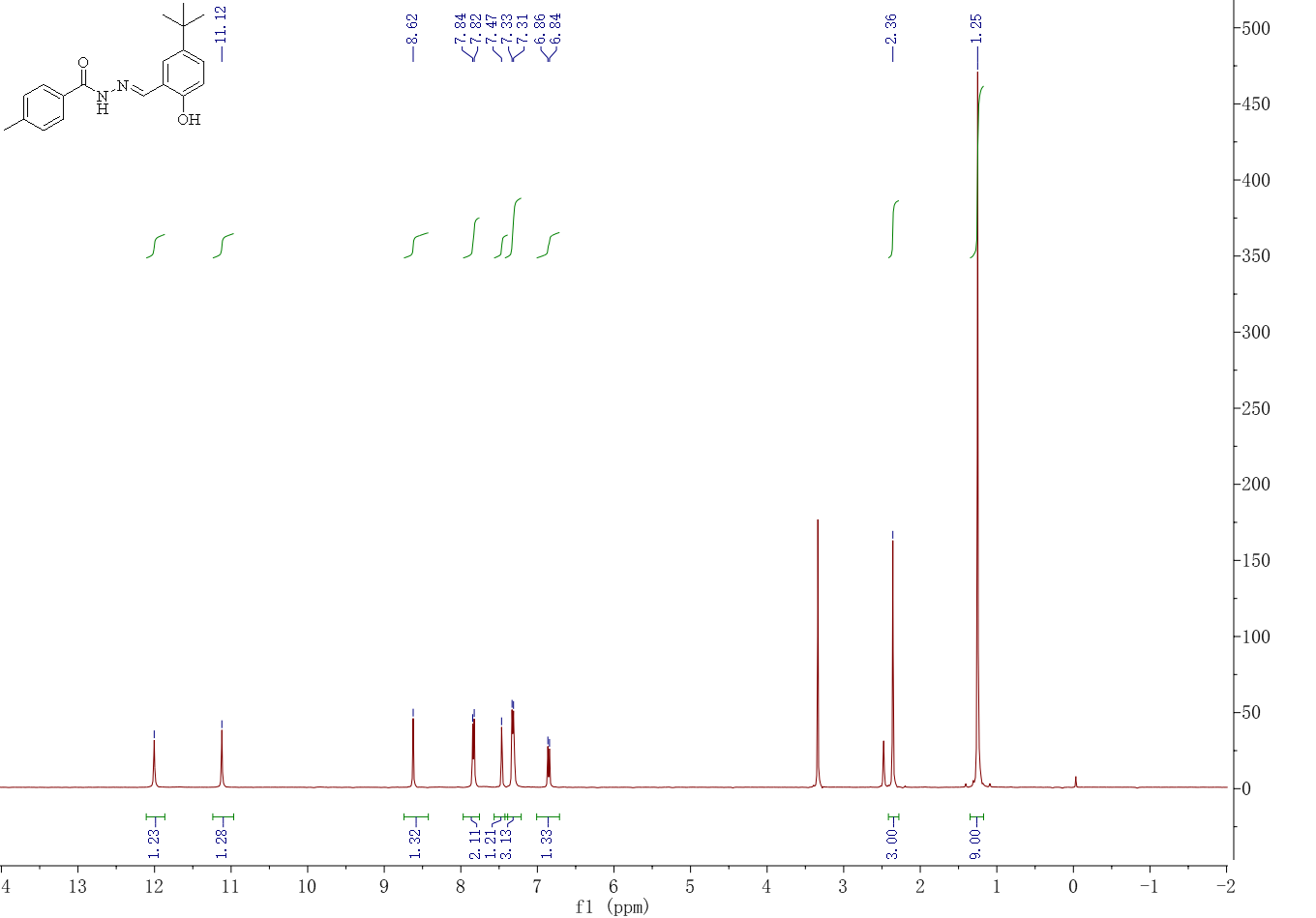


Fig 25. *^1^H NMR of* **C13** (400 MHz, DMSO)


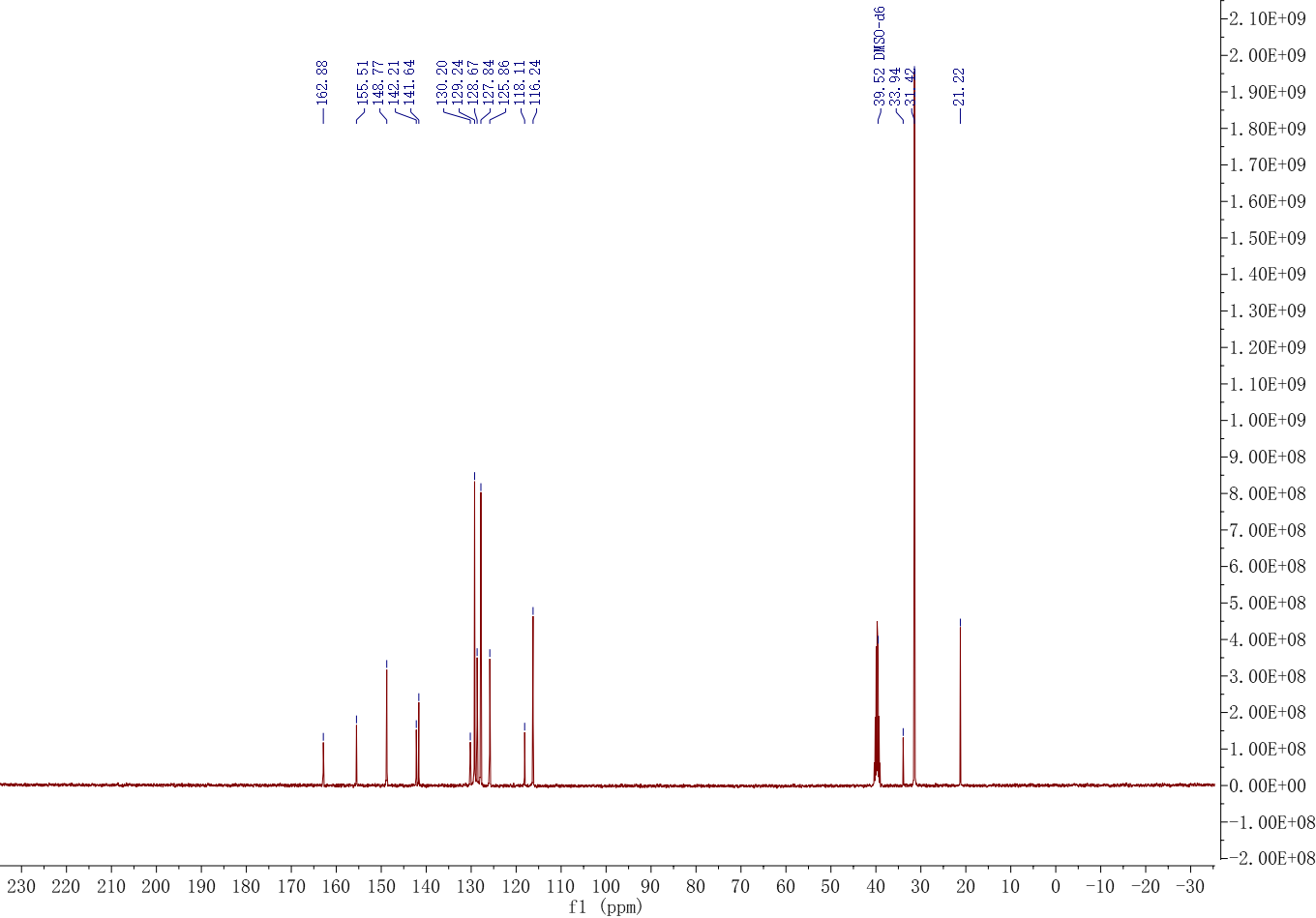


Fig 26. *^13^C NMR of* **C13** (100 MHz, DMSO)


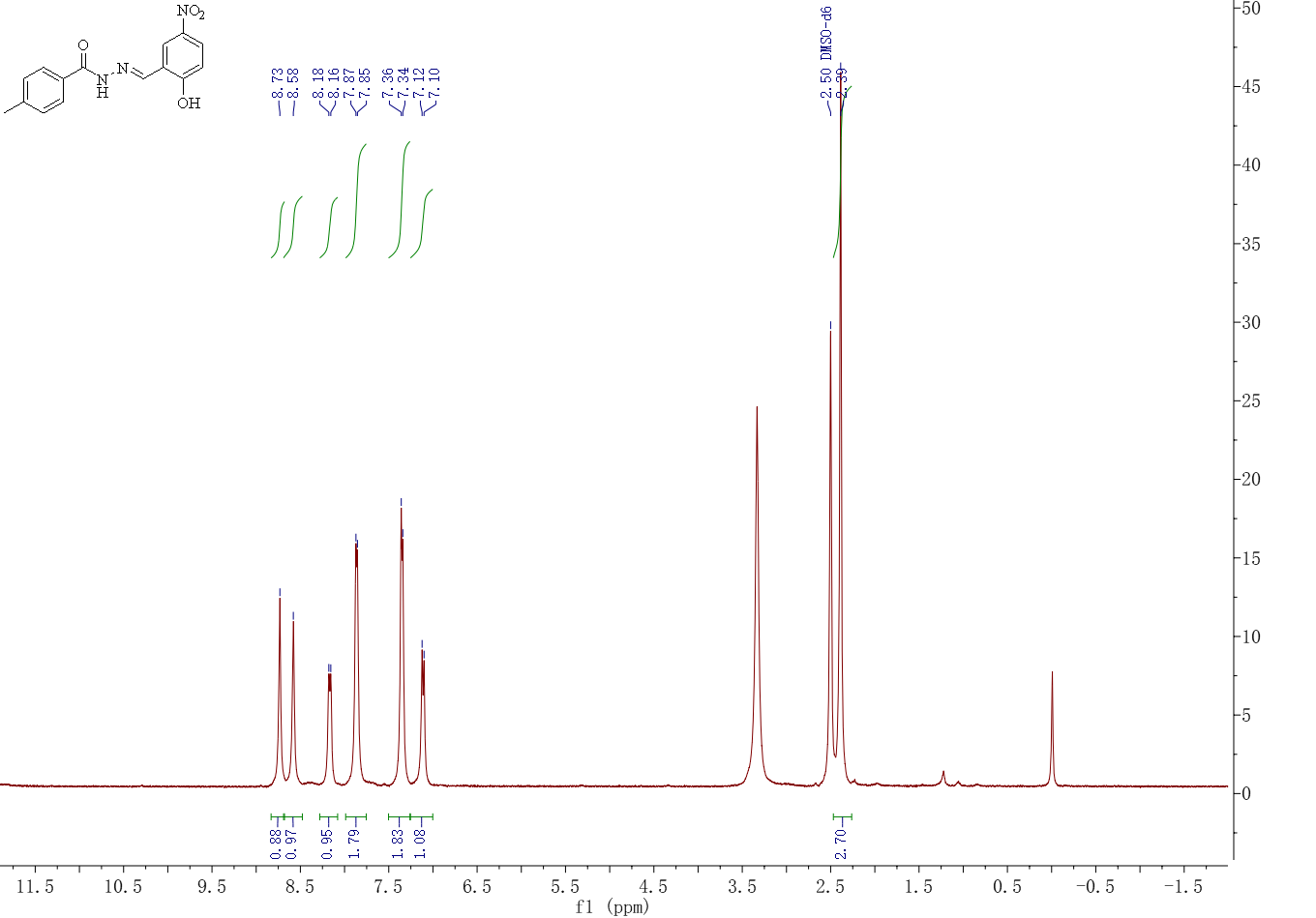


Fig 27. *^1^H NMR of* **C14** (400 MHz, DMSO)


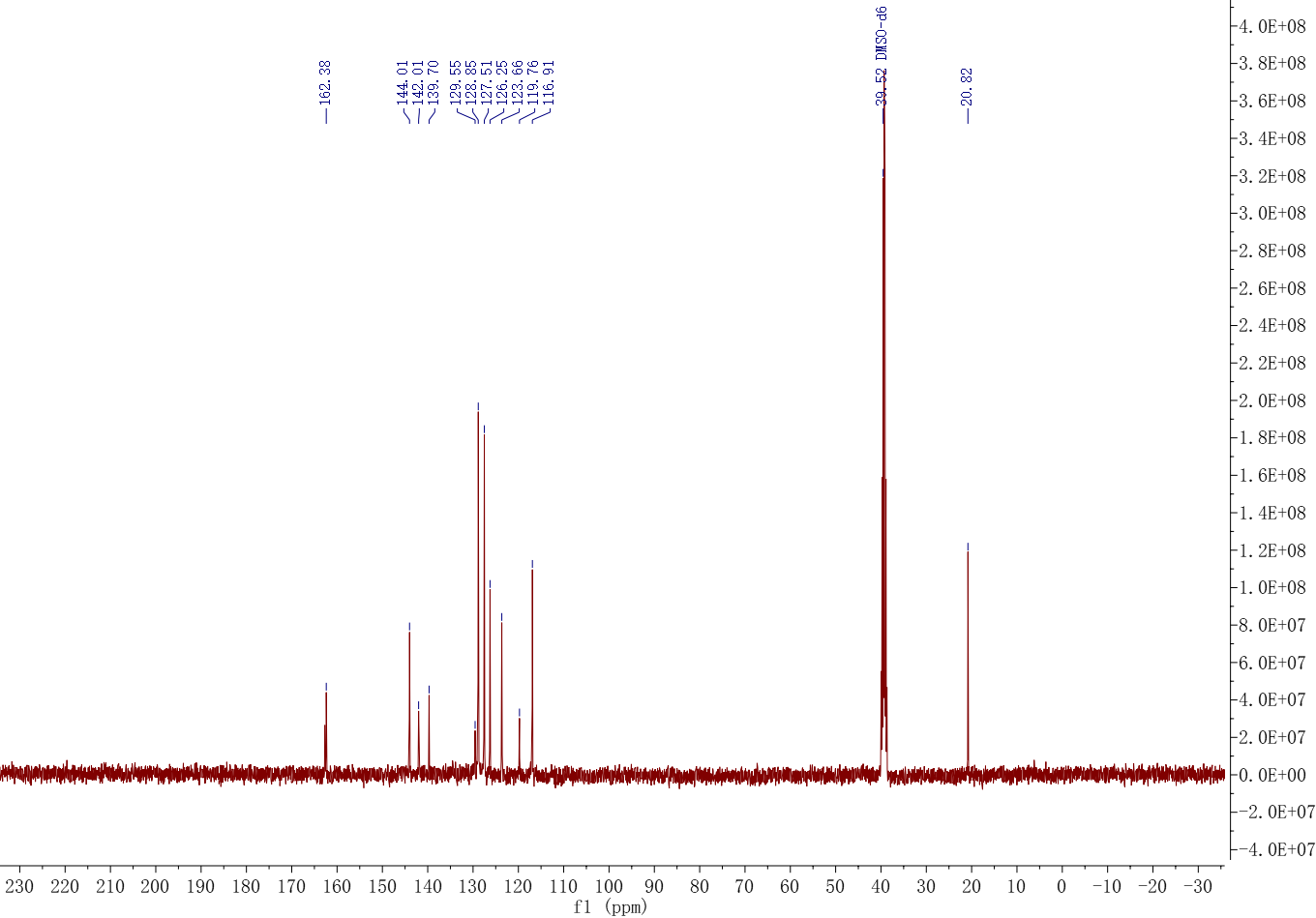


Fig 28. *^13^C NMR of* **C14** (100 MHz, DMSO)


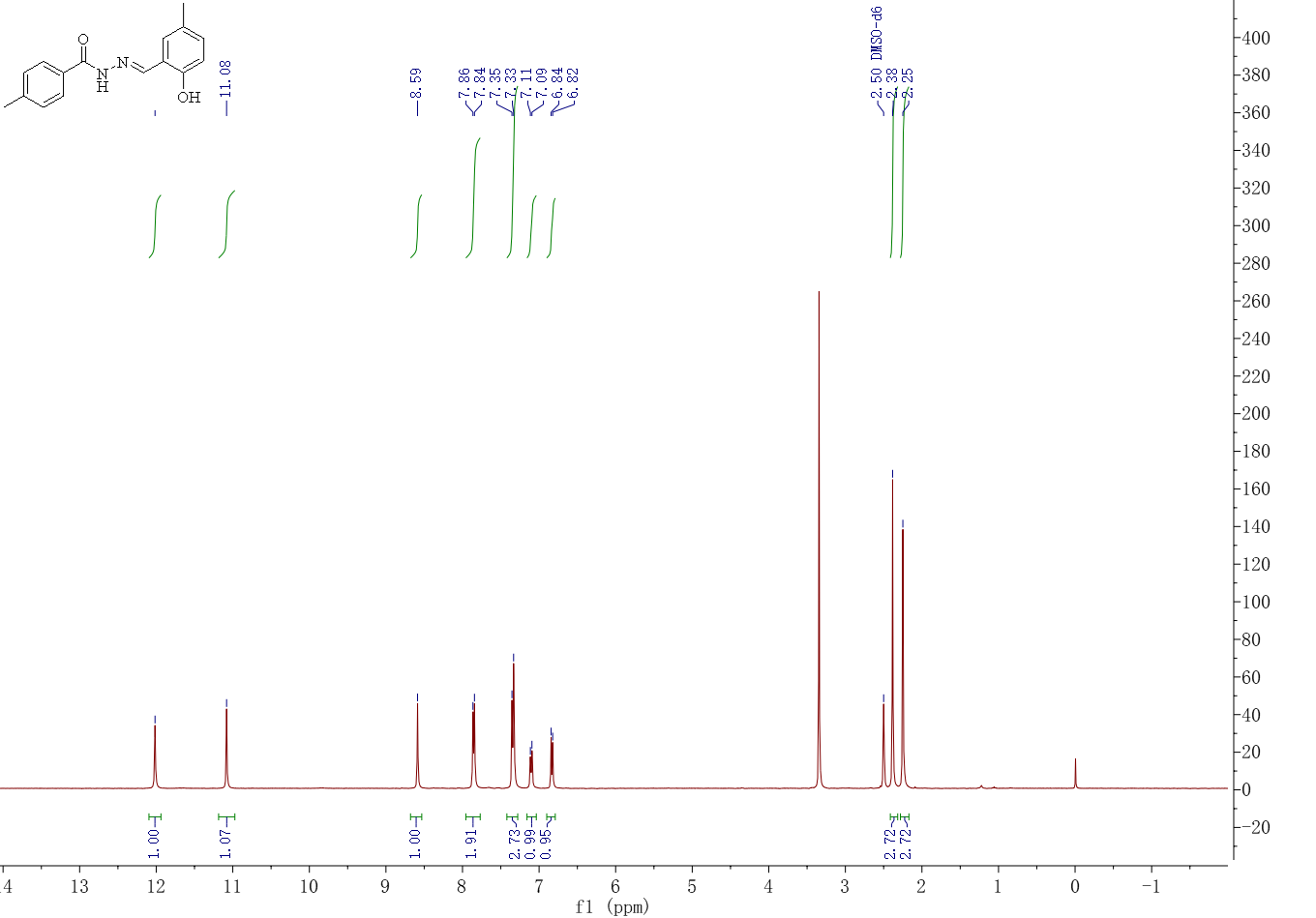


Fig 29. *^1^H NMR of* **C15** (400 MHz, DMSO)


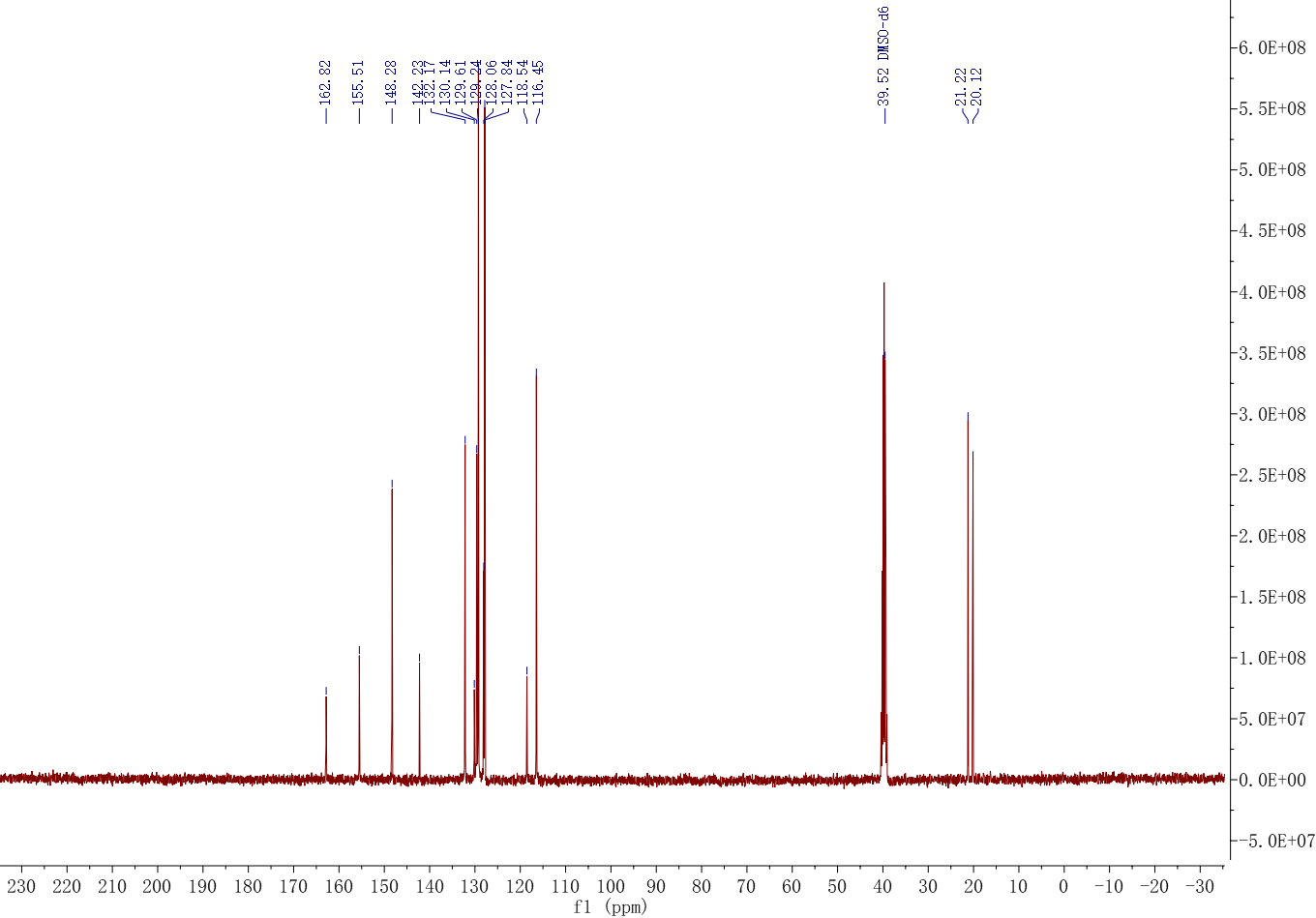


Fig 30. *^13^C NMR of* **C15** (100 MHz, DMSO)


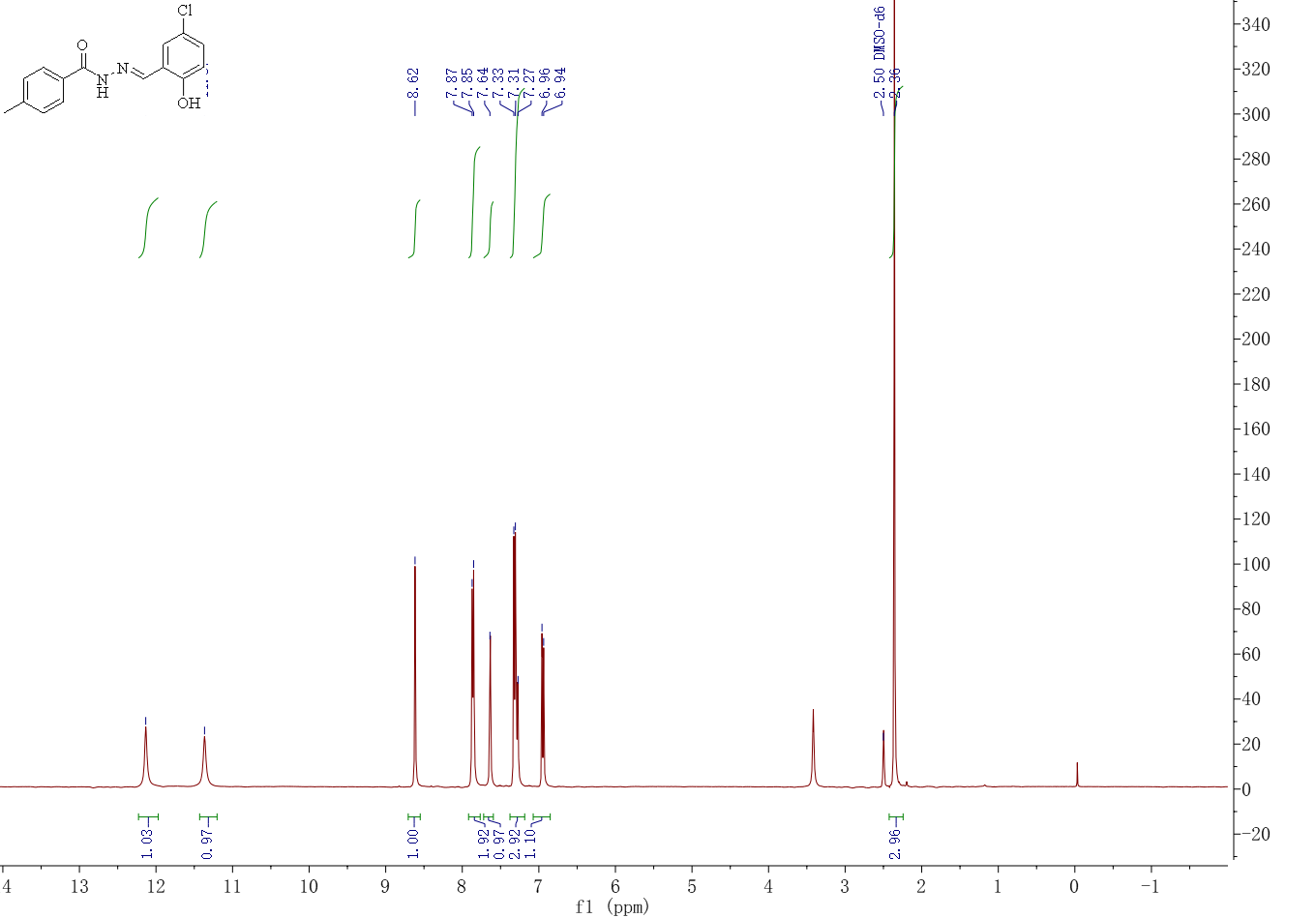


Fig 31. *^1^H NMR of* **C16** (400 MHz, DMSO)


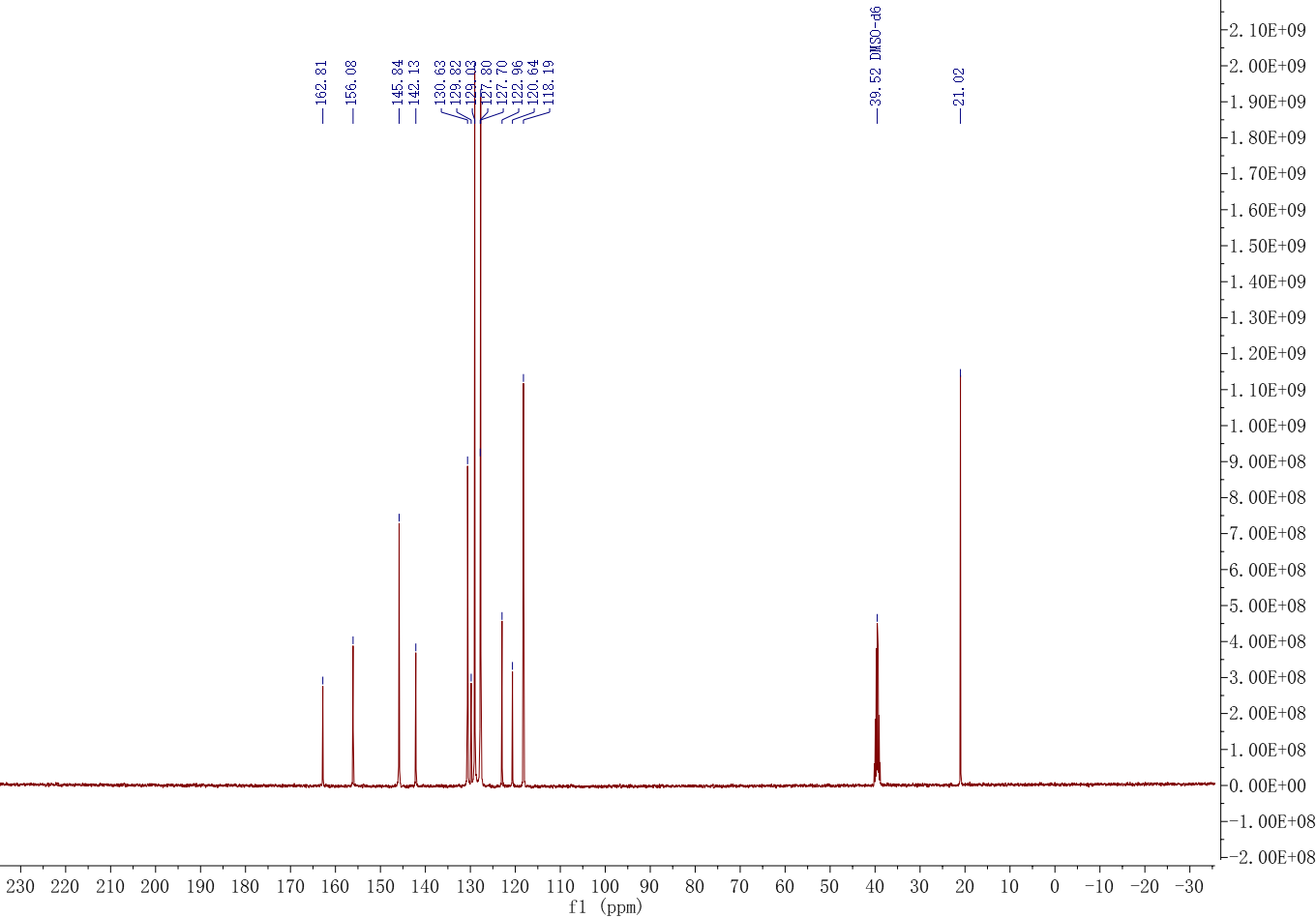


Fig 32. *^13^C NMR of* **C16** (100 MHz, DMSO)


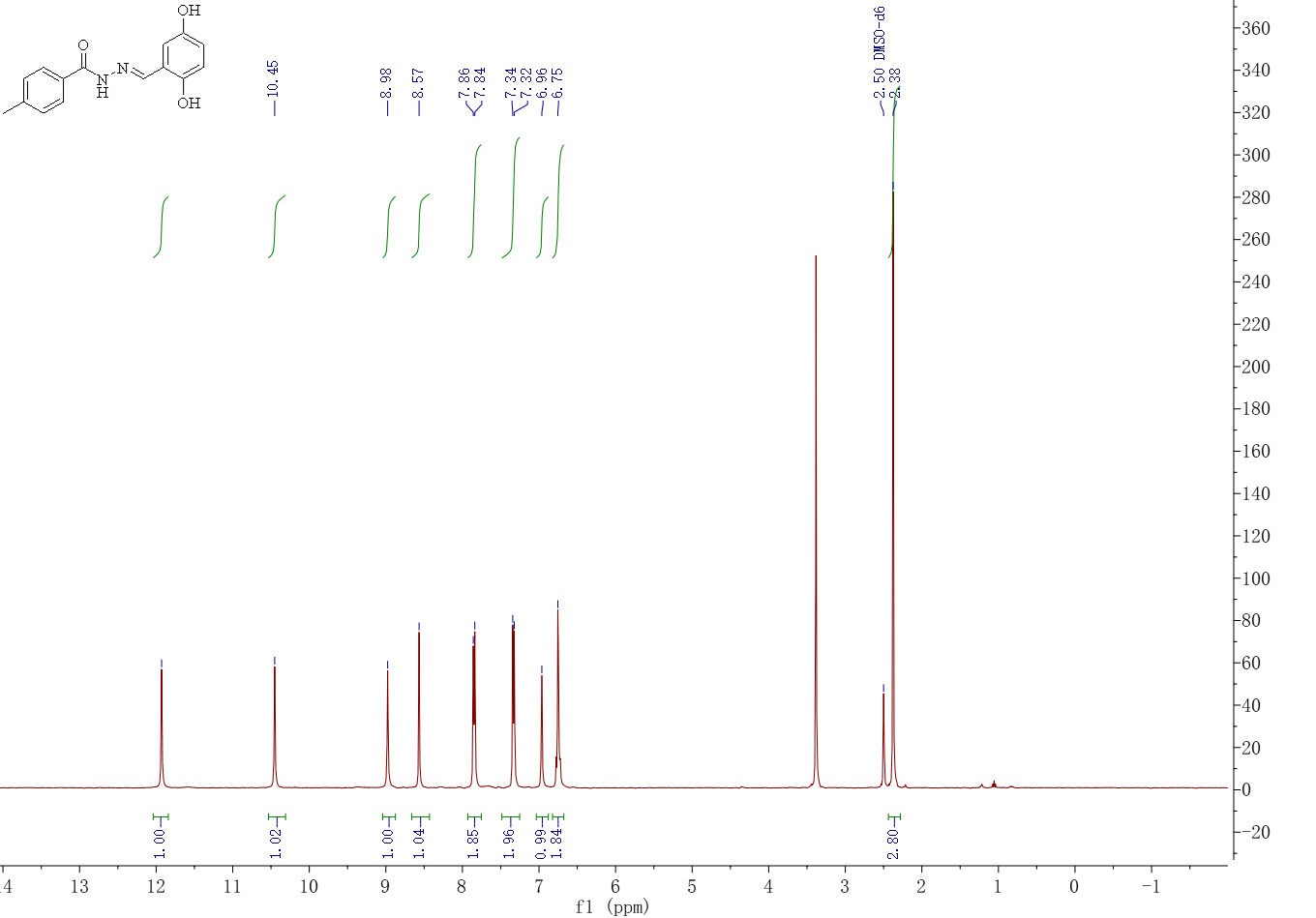


Fig 33. *^1^H NMR of* **C17** (400 MHz, DMSO)


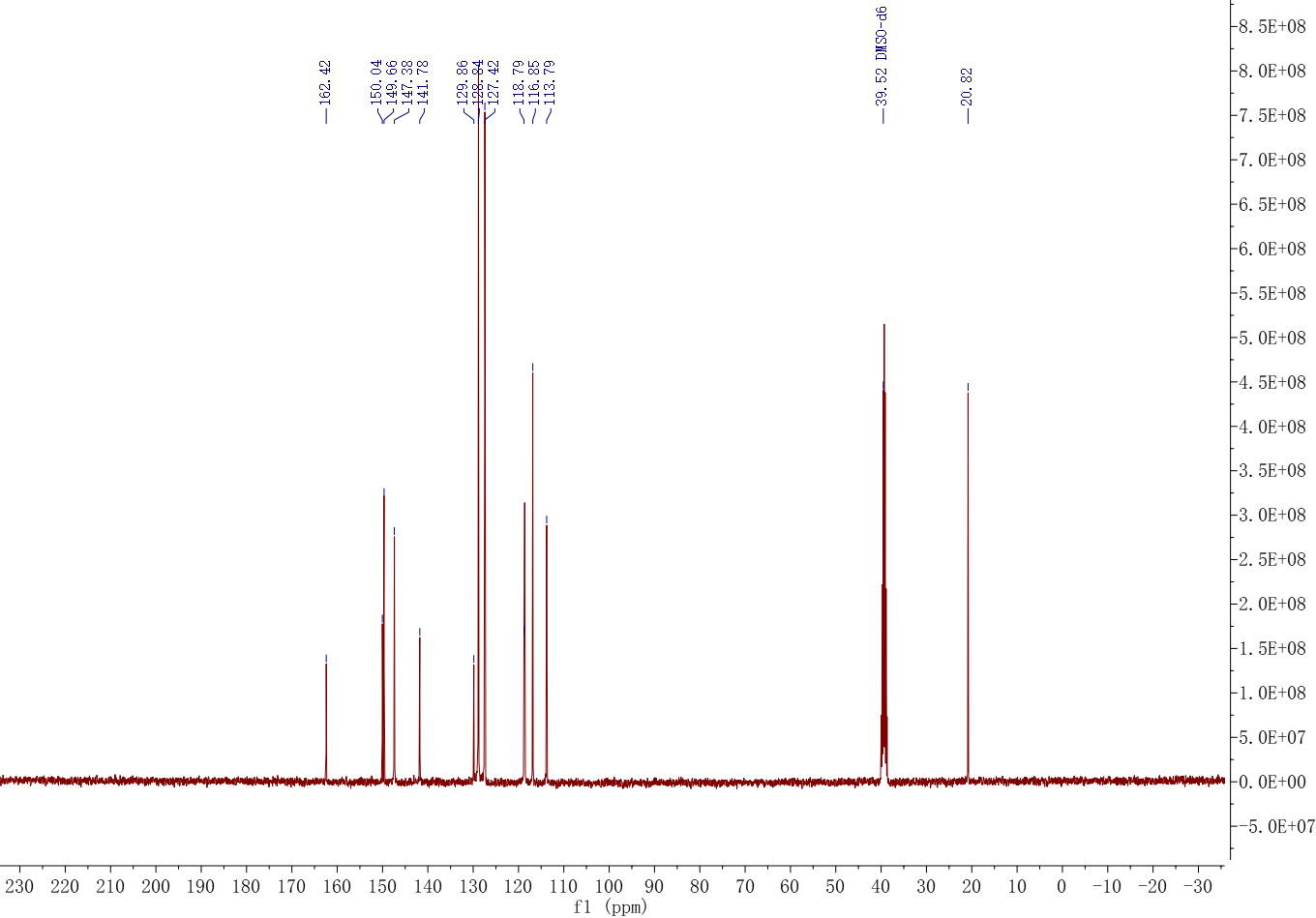
Fig 34. *^13^C NMR of* **C17** (100 MHz, DMSO)


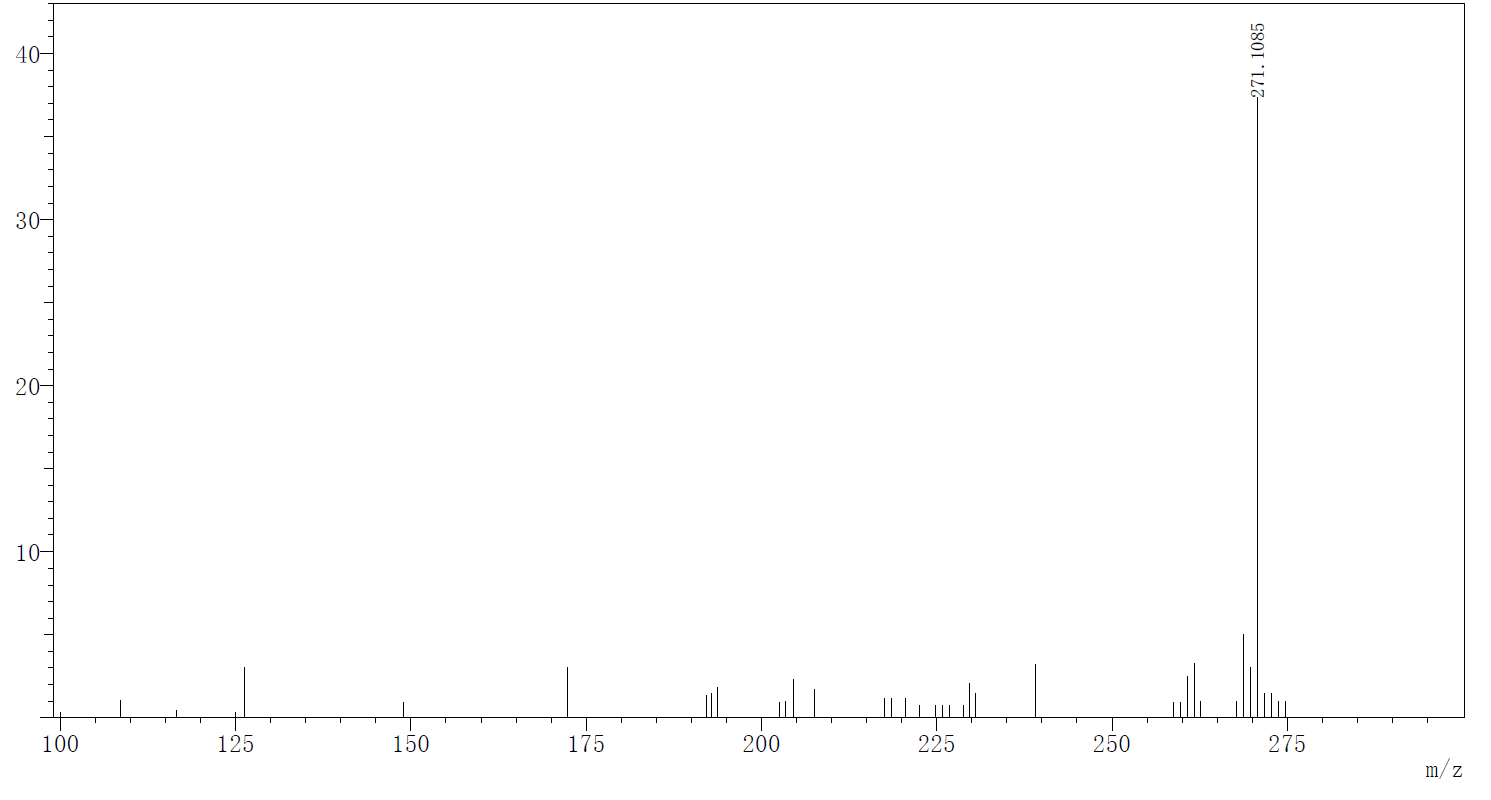


Fig 35. *Mass spectrum of* **C17**


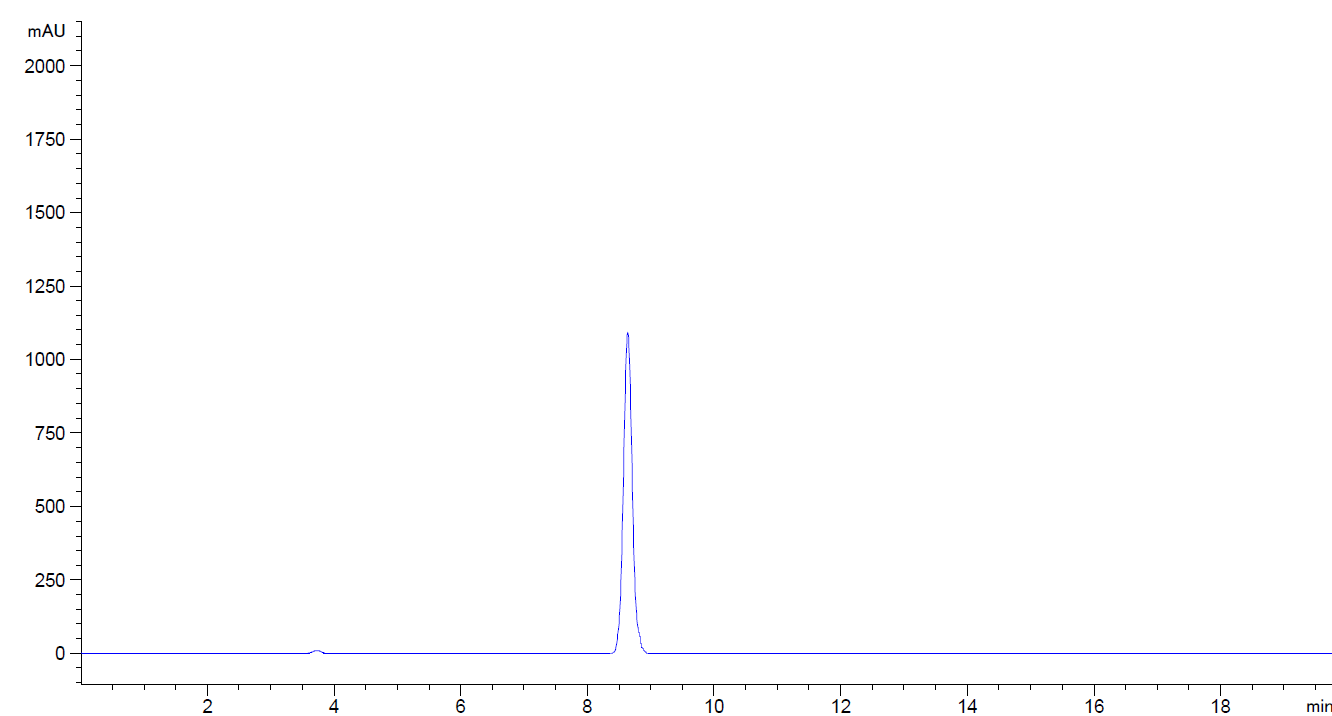


Fig 36. *HPLC purity chromatogram* *of* **C17**


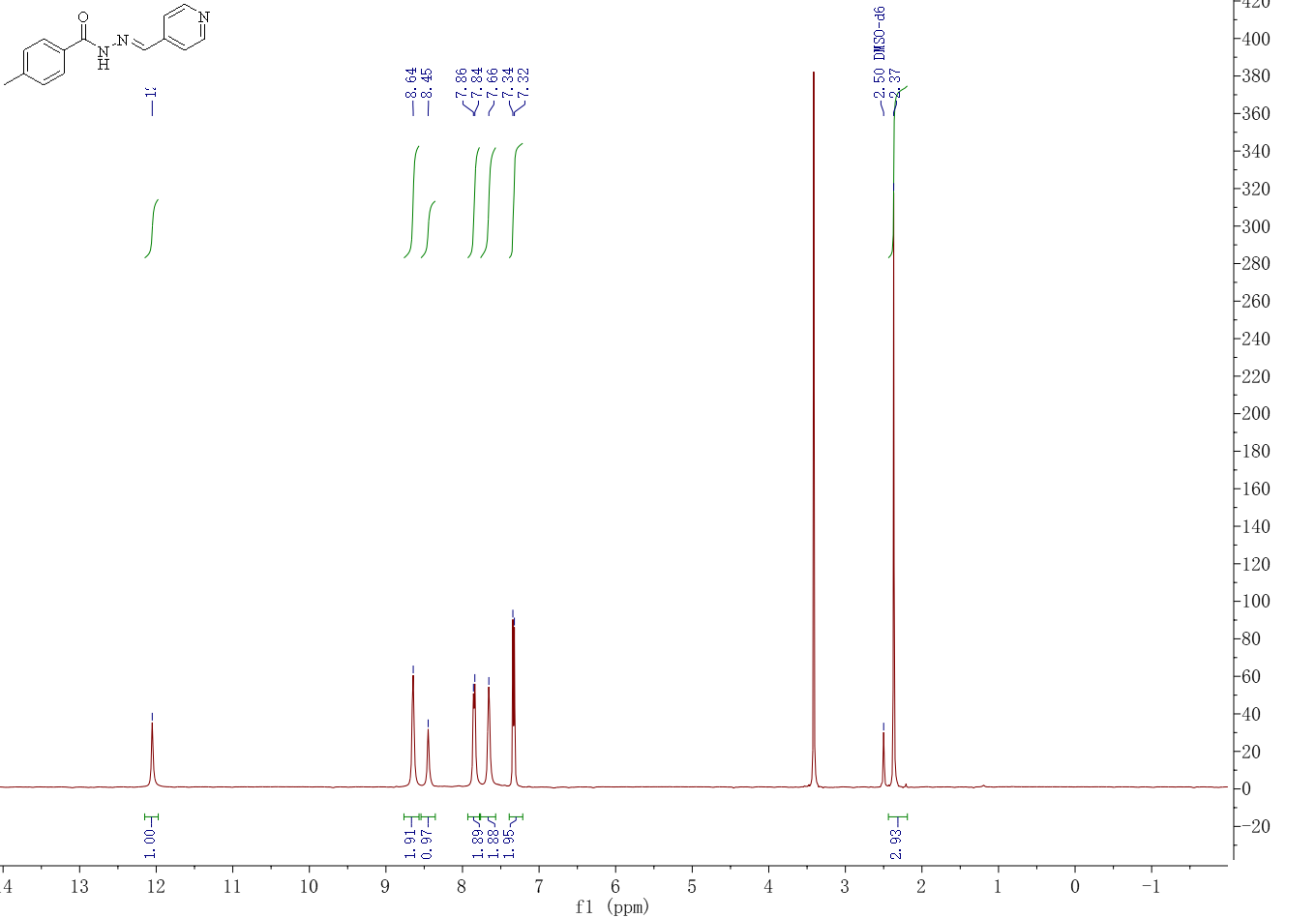


Fig 37. *^1^H NMR of* **C18** (400 MHz, DMSO)


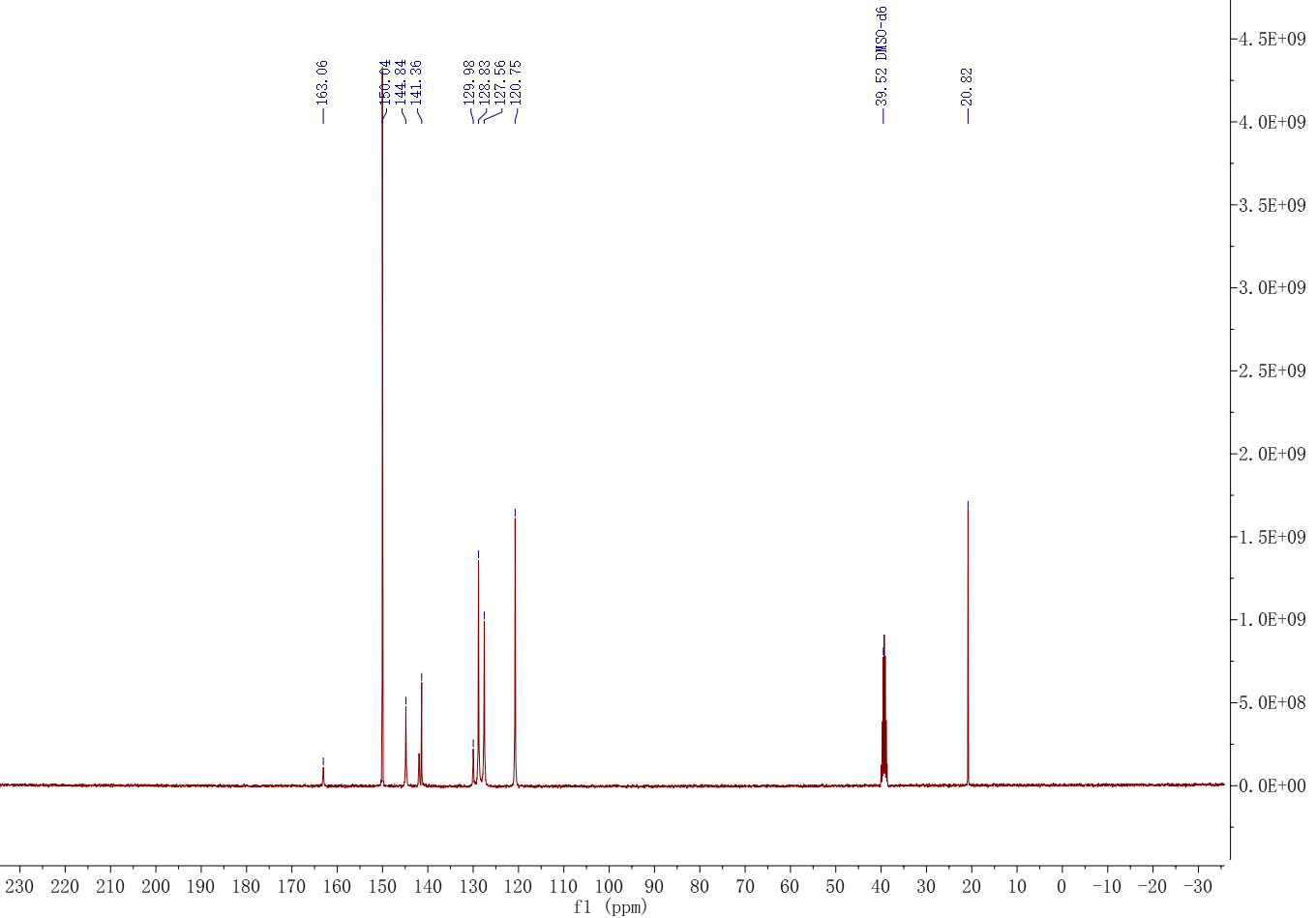


Fig 38. *^13^C NMR of* **C18** (100 MHz, DMSO)

1. *Correspondent. E-mail: [h418561754@163.com](mailto:h418561754@163.com) [↑](#footnote-ref-2)
